# Exploring the regional layout characteristics of ancient Chinese postal system in coastal areas based on AHP-CRITIC evaluation approach

**DOI:** 10.1371/journal.pone.0333348

**Published:** 2025-09-25

**Authors:** Bei Wu, Lifeng Tan, Chang Cui

**Affiliations:** 1 Institute of Architectural History, China Architecture Design and Research Group, Beijing, China; 2 School of Architecture, Tianjin Chengjian University, Tianjin, China; 3 Beijing Urban Construction Design and Development Group, Beijing, China; Universiti Teknologi Malaysia - Main Campus Skudai: Universiti Teknologi Malaysia, MALAYSIA

## Abstract

In ancient China, as the only transmission network, the postal system enabled long-distance delivery of information and supplies, providing information support for the development of ancient society. Delivery efficiency determined whether information and supplies could arrive at the fastest speed, reflected the level of regional transmission capacity, and served as an effective indicator for understanding the underlying logic of the postal system’s regional layout. In this study, the concept of accessibility was introduced into the regional layout analysis of the postal system, and a hierarchical evaluation model was constructed based on the AHP-CRITIC (Analytic Hierarchy Process – Criteria Importance Though Intercriteria Correlation) composite weighting method. Then, the accessibility of the postal system in three typical areas of coastal defense front-line, transportation hub, and administrative center was qualitatively and quantitatively analyzed, and the differential characteristics of their accessibility and regional layout were summarized, so as to explore the layout logic of the postal system during the Ming Dynasty.

## 1 Introduction

Ancient civilization centers, such as China, Egypt, Assyria, Persia, Rome, Greece, and Arabia, left a rich heritage in human history, and also contributed to the development of transportation. [1] The ancient postal system can be traced as far back as Ancient Greece (around 2,000 BC) and the Shang Dynasty (1,600 BC – 1,046BC) in Ancient China, when messengers specialized in transmitting messages already emerged [2]. With the evolution of civilization and the advancement of tools, the relay first appeared in China and developed vigorously in the 7th and 8th century BC. Meanwhile, the great Persian Empire of Cyrus in the 6th century BC also employed relays of mounted messengers. [1] Relay was a way of transmission from one station to the next along a predetermined route. Due to its high efficiency, convenience and unified management, relay became the most common and continuously used transmission form of the ancient postal system, and left an indelible impact on modern postal services. A systematic study of the ancient postal system not only matters a lot to the preservation of postal heritage, but also secures a deeper understanding of the history of the post as an essential infrastructure for human life.

The Ming Dynasty witnessed the high development of the ancient Chinese postal system, and also marked the last period of prosperity before new postal services prevailed. The Ming postal system relied on 3 types of delivery facilities: Yizhan (驿站), commonly located along the major roads or waterways of the empire for delivering official documents, military reports and supplies; Diyunsuo (递运所), set up only in some important areas and dedicated to the transportation of goods; Jidipu (急递铺), the most commonly seen delivery facilities set up every 10 miles only for the couriers to deliver documents by walking. [3] All the facilities sought to maximize delivery efficiency by breaking down a long journey into many shorter segments, making the postal system function in a systematic form. As an effective tool for the Ming government to strengthen the centralized rule, the postal system not only promoted the dissemination of decrees and the economic exchanges across the country, but also played a key role in transmitting military situations and delivering supplies, serving as an indispensable component of the defense system and providing important historical and military value. [4] Although the Ming postal system has been extensively and thoroughly explored, there’s a trend of multi-polarized development. On the one hand, research on the development history of the postal system [2,5,6], the transportation carriers [7–9] and their economic value [10] has achieved fruitful results. On the other hand, some basic data about the Ming postal system has not yet been figured out, including its network and layout characteristics. Generally speaking, the existing studies mostly focused on historical and qualitative perspectives, but lacked analysis of the overall spatial structure and layout mechanism. As the research on the ancient postal system goes deeper, these issues urgently need to be addressed.

Similar to modern postal services, the ancient postal system functioned as a system, and how its regional layout was planned exerted a direct impact on the transmission level. Specifically, a highly extensible network is vital to the postal system. The widespread postal facilities brought about great accessibility and a high level of transmission. It must be noted that this influencing factor is space-related, but not correlated with capital or technology. Accordingly, to systematically understand the regional layout characteristics and key areas of the ancient postal system, “accessibility” can be a good criterion.

The concept of accessibility has existed for a long time to mainly reflect the difficulty level of spatial activities from diverse perspectives [11], especially in the fields of human geography, geo-information science, urban planning, transportation planning, and regional economic analysis. The study on accessibility originated from classical location theories. In Thunnen’s agricultural location theory [12] and Weber’s industrial location theory [13], accessibility was used as an indicator to reveal transportation costs. As a flexible concept, accessibility is assigned with different connotations and calculation measures by scholars to tackle different practical problems. In Hansen’s potential model [14], accessibility was defined as the level of opportunity to access a given place, for the purpose of exploring urban land use. Ingram [15] measured accessibility based on the degree of spatial barrier by leveraging indicators like distance, time and costs, and suggested a negative correlation between spatial barrier and accessibility. Wachs and Kumagai [16] saw accessibility as the opportunities obtained per unit of time or space, which are positively related. Vickerman [17] defined accessibility as the topological distance between nodes. Although the concept of accessibility has not been conclusively defined, scholars generally agree that accessibility can be understood as the convenience degree of movement between designated two designated points in the transportation system, depending on the mobility of individuals and the number of opportunities. [18] By pairwise comparison of accessibility, the location advantage of a certain point in the global network can be reflected. Accordingly, accessibility is increasingly being used to evaluate the coverage and layout of regional transportation networks and infrastructures, such as subway system [19], cycle network [20], public transport [21], urban services for the elderly [22], urban green infrastructure [23], etc.

Accessibility analysis works efficiently not only for modern urban research but also for cultural heritage research. Lin Zurui et al. [24] discussed the accessibility characteristics of public spaces in traditional villages through kernel density analysis. Lin Zhisen et al. [25] used accessibility to analyze the traffic and sight line of military settlements in Minjiang Estuary, so as to summarize the rescue characteristics. Yin Zekai et al. [26] applied accessibility to coastal defense research, discussing the concepts and associated features of accessibility on visual, auditory, traffic, and weapon in military elements. These findings enriched the perspectives of cultural heritage research and provided valuable references for future studies. However, in terms of methodology, qualitative analysis still accounted for a significant proportion. In this sense, although relatively credible results have been achieved, accessibility is not comprehensively evaluated, and the correlation between spatial features is not thoroughly discussed, leading to less objective conclusions. Hence, new approaches and data support are needed to spatially and visually characterize the accessibility of the ancient postal system.

Given the issues highlighted above, the study design is outlined as follows:

**Aim**: Address the current gap regarding the comprehensive spatial structure and layout mechanisms of the ancient postal system, thereby advancing scholarly understanding in this field.

**Objectives**: Construct an applicability evaluation system for accessibility by integrating distinctive characteristics of ancient postal systems with existing applicability evaluation methods to assess regional accessibility levels, identify spatial distribution patterns and prioritize areas, and then analyze factors influencing the spatial configuration mechanism of the system.

**Hypothesis**: Under the influence of different geographic locations, function attributes, and terrain characteristics, the layout priorities of ancient postal system varied and were potentially linked to the corresponding historical background.

**The outcomes and contributions**:

1) Spatiotemporal reconstruction: Based on historical documentation, the spatial distribution of postal system in three regions (Wenzhou Prefecture, Tingzhou Prefecture, and Guangzhou Prefecture) were visualized, and their transmission networks were reconstructed.
2) Evaluation framework development: A hierarchical accessibility assessment framework was established with three dimensions and four factors. The AHP-CRITIC (Analytic Hierarchy Process – Criteria Importance Though Intercriteria Correlation) composite weighting method integrates subjective and objective weighting approaches to enhance evaluation reliability.
3) Mechanism analysis: Quantitative spatial analysis revealed regional characteristics and influencing factors of the postal system’s layout, while exploring its contributions to state governance and societal development in historical China.

## 2 Methods and materials

**Fig 1:**
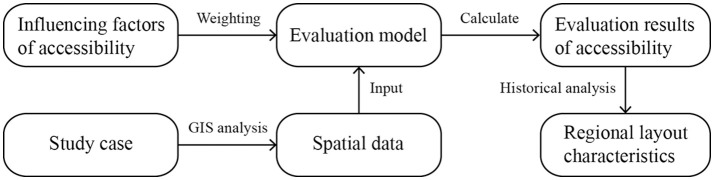
Technical route of the study.

The technical route of this study is shown in Fig 1. First, influencing factors on the accessibility of the postal system were systematically analyzed to establish an accessibility evaluation model for the postal system. Second, focusing on several representative coastal areas, the database was built using the ArcGIS platform, and spatial data of postal facilities corresponding to the evaluation indicators were extracted accordingly. Third, the available spatial data were input into the evaluation model for calculation; the accessibility results of each region were then spatially projected to generate a spatial distribution map of accessibility. Finally, through inter-regional horizontal comparisons, subtle differences in accessibility were spatially presented, and the underlying drivers of these differences were identified by combining historical context and geographical environment characteristics.

### 2.1 Hierarchical evaluation model

The accessibility of the postal system is crucial to coastal warfare. For example, if a coastal area faces a large-scale attack and the garrison of nearby forts can only hold out for several days, a distress signal must be transmitted to other forts through the postal system. With high postal accessibility, surrounding forts can receive the information and dispatch troops to reinforce and defend the threatened coastal area in the shortest time; otherwise, the area may fall. For this reason, governments prioritized measures to enhance postal system accessibility in key regions during planning.

In the context of postal systems, accessibility centers on measuring the transmission efficiency and service coverage across spatial locations through this network. Drawing on existing studies, the accessibility of postal system can be deconstructed into three interconnected core dimensions: timeliness, connectivity, and coverage (Table 1). Timeliness focuses on maximizing transmission speed and minimizing time consumption. Connectivity measures spatial connection strength and path diversity between network nodes. Coverage evaluates the effective geographic scope of postal services reaching different locations within a region. Collectively, these three dimensions form the essential components for understanding the accessibility of ancient postal systems.

**Table 1:** Three dimensions of the postal system’s accessibility.

Dimension	Definition	Sources
Timeliness	Time/cost efficiency to specific destinations	Hansen (1959) [14]; Ingram (1971) [15]
Connectivity	Connection strength and path diversity between nodes in the spatial network	Kansky (1964) [27]; Derrible, Kennedy (2010) [28]
Coverage	Spatial coverage and equity of services	Shen (1998) [29]; Geurs, Ritsema van Eck (2001) [30]

Factors influencing postal system performance across the three core dimensions collectively shaped transmission accessibility. By analyzing the attributes and transmission process of the postal system, four factors were summarized, as shown in Table 2.

**Table 2:** Influencing factors of the postal system’s accessibility.

Influencing factor	Statement
Number of the postal facilities	Essential for improving coverage and connectivity. Higher facility density reduces service gaps and increases path options and redundancy among nodes.
Geographical conditions (elevation, slope and relief)	Decisive for timeliness. High elevation, rugged terrain, and steep slopes typically create substantial transmission barriers by significantly reducing speed while increasing physical exertion and risks, and consequently diminishing timeliness performance.
Distance between postal facilities	Critical to connectivity and timeliness. Relay transmission is to divide a transmission process into discrete segments. Longer distances between neighboring nodes generally increase time costs per relay, directly undermining connectivity and timeliness across the transmission process.
Jurisdiction area	Jurisdiction area represents the spatial scope serviced by individual facilities, which is a key factor affecting coverage while simultaneously constraining timeliness. Larger jurisdictions increase transmission pressure and prolong delivery time from peripheral areas, ultimately restricting timeliness across the entire transmission process.

Notably, these four fundamental factors do not operate in isolation but collectively shape the three core dimensions through dynamic interactions. The number of postal facilities and jurisdiction area are closely linked, typically exhibiting an inverse relationship (increased facility quantity usually corresponds to reduced jurisdiction size), jointly determining the coverage level and spatial distribution uniformity of the network. The distance between postal facilities is strongly correlated with jurisdiction area, as jurisdictional boundaries are often delineated based on distances to neighboring facilities. Ideal postal accessibility represents the comprehensive optimization of multiple factors: an adequate number of facilities (to ensure coverage and connectivity), appropriate inter-facility distances (to secure connectivity and timeliness), compact jurisdiction size (to minimize internal response time), and favorable topography (to enhance travel efficiency).

Having defined the accessibility dimensions and influencing factors of postal systems, the next step is to systematize and hierarchize these influencing factors according to the structural composition of the postal system, and then construct an ideal evaluation framework.

Based on the postal system’s composition, the accessibility of Yizhan, Diyunsuo and Jidipu can be analyzed individually, with each contributing collectively to the overall accessibility of the postal system. Subsequently, the attributes of Yizhan, Diyunsuo and Jidipu are integrated with the general influencing factors outlined in Table 2 to determine the specific evaluation indicators.

**Fig 2:**
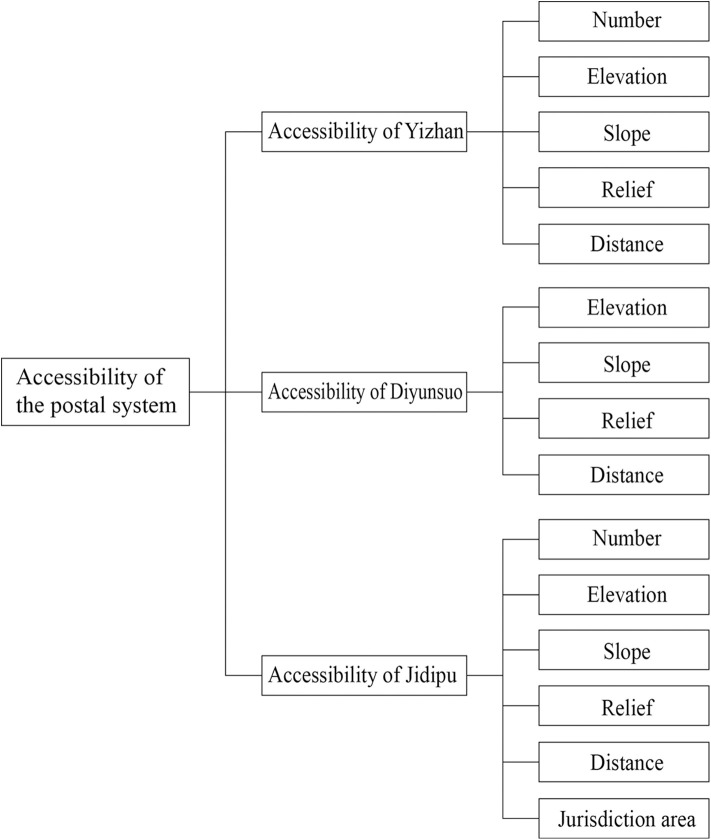
Evaluation framework for the accessibility of the postal system.

Furthermore, the applicability of uniform evaluation indicators to Yizhan, Diyunsuo, and Jidipu requires exploration. Given that this study adopts counties, the smallest administrative units governing the postal system, as the basic unit for discussing regional layout characteristics, while Yizhan served prefectural-level administrative areas and Diyunsuo primarily handled provincial-level goods transportation. Relatively, although Yizhan and Diyunsuo were geographically situated within counties, their delivery tasks and service scope extended beyond county boundaries. Thus, the jurisdictional area of Yizhan and Diyunsuo cannot be used as an evaluation indicator for accessibility. Additionally, Diyunsuo were established in only a small number of counties, with merely one per county; this makes comparing their quantity an infeasible method for measuring accessibility. In contrast, Jidipu were numerous and widely distributed, serving as the core component of the county-level postal system and extending to areas beyond the reach of Yizhan and Diyunsuo. Therefore, all identified influencing factors should be considered when measuring Jidipu accessibility. Following this analysis, an evaluation framework for the postal system’s accessibility can be constructed, as shown in Fig 2.

### 2.2 Composite weighting method AHP-CRITIC

Since the significance of each indicator in the evaluation framework varies, so that it is necessary to weigh their relative importance. The weighting methods can be categorized into two types: subjective and objective weighting methods. For subjective weighting methods like Delphi and AHP methods, indicator weights are calculated based on subjective qualitative analysis and judgment. In contrast, objective weighting methods determine weights through statistical analysis of the indicator data’s variation degree, including entropy method, coefficient of variation (CV) method and CRITIC method. [31] Different methods involve distinct mathematical calculations, leading to variations in the resulting weights. Thus, an appropriate weighting method shall be selected.

In the evaluation framework of the postal system’s accessibility, first-level indicators are categorized based on the postal system’s composition (Yizhan, Diyunsuo, and Jidipu) and lack data attributes, so only subjective weighting methods are applied here. Second-level indicators, by contrast, correspond to objective factors with extractable data, necessitating both subjective and objective weighting methods to calculate their weights. To avoid result deviations caused by extreme data, a combination of subjective and objective weighting methods is used to determine the weights of second-level indicators.

Among subjective weighting methods, the AHP method integrates qualitative analysis with quantitative computation. It involves pairwise comparisons of indicator importance through subjective judgment, followed by mathematical derivation of weights, making it a scientifically robust subjective weighting approach. In contrast, Delphi method requires recruiting domain experts as evaluators, relying entirely on their individual experience. This approach not only demands high professional competence and comprehensive understanding from evaluators but also faces challenges in ensuring consistency among their perspectives, often leading to less ideal assessment outcomes. [32] For this reason, AHP method is more suitable for calculating indicator weights in the postal system’s accessibility evaluation framework.

Among the objective weighting methods, both the entropy method and CV method calculate weights based on the degree of variation between indicators, whereas the CRITIC method considers both variation and correlation [31]. As previously noted, factors such as the number of postal facilities, inter-facility distance, and jurisdiction area exhibit strong correlations; similarly, geographical evaluation indicators (elevation, slope, and relief) are typically interrelated. The CRITIC method can effectively neutralize such correlations and reduce the weights of interrelated factors, rendering the final results more scientific. Consequently, the CRITIC method is more suitable for analyzing the accessibility of the postal system.

**Fig 3:**
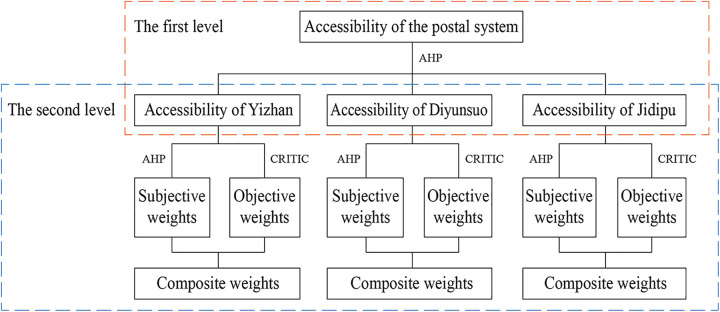
Technical route of composite weighting method AHP-CRITIC.

In light of the above considerations, the AHP-CRITIC composite weighting method is adopted to calculate the weight of each indicator, with the corresponding technical route shown in Fig 3. In general, the AHP method effectively processes subjective information, while the CRITIC method extracts objective information from research data. Their integration balances both subjective and objective dimensions. Recognized for ensuring the validity and reliability of derived weights, this composite approach has been increasingly applied in assessment studies across diverse fields in recent years [33–35].

#### 2.2.1 AHP method.

The AHP method includes qualitative analysis and mathematical calculation. The specific steps are as follows [36]:

1) Constructing the judgment matrix. By pairwise comparing the importance of indicators in the same set relative to the corresponding indicators at the upper level, and using the numbers 1–9 and their reciprocal as standardized metrics, the judgment matrix is constructed. [37]
2) Calculating the weight values. Calculate the n-th root $\alpha_{\text{i}}$ of the product of each row figures in the judgment matrix, where n is the number of indicators, and then normalize the vector $\overset{―}{\alpha_{\text{i}}}=(\overset{―}{\alpha_{\text{1}}},\;\overset{―}{\alpha_{\text{2}}},\;\overset{―}{\alpha_{\text{3}}})$ by using the following equation.

$$\alpha_{i}=\frac{\overset{―}{\alpha_{i}}}{\sum_{i=\text{1}}^{n}\overset{―}{\alpha_{i}}}$$ (1)

$\alpha_{\text{i}}$ is the weight value.

3) Consistency test. To ensure the rationality of indicator weights, it is necessary to calculate the CR value and CI value, and then conduct a consistency test. The testing formula is as follows:


$$\text{CR}=\frac{\text{CI}}{\text{RI}}$$ (2)

$$\text{CI}=\frac{\lambda_{\text{max}}-\text{n}}{\text{n}-\text{1}}$$ (3)

$$\begin{array}{c} \lambda_{max}=\sum {\mathrm{\backslash nolimits}}_{i=i}^{n}\frac{{\left(A\alpha \right)}_{i}}{n\alpha_{i}} \end{array}$$ (4)

where λ_max_ is the maximum eigenvalue, RI is the average random consistency index, given by fixed values. [37]

If CR < 0. 1, or if λmax = n and CI = 0, it is considered that the judgment matrix has an acceptable consistency and the weight values take effect. Otherwise, the importance values in the judgment matrix should be adjusted.

#### 2.2.2 CRITIC method.

The CRITIC method calculates weights based on statistical analysis of data. The specific steps are as follows [38]:

1) Data normalization processing. To avoid the impact of numerical differences on the analysis results, it is necessary to normalize the data of each indicator and limit them to a certain range ([0,1] or [−1, 1]). The formula is as follows:

$${\text{x}}_{\text{ij}}^{\prime }=\frac{x_{ij}-x_{j}^{min}}{x_{j}^{max}-x_{j}^{min}}$$ (5)

where ${\text{x}}_{\text{j}}^{\text{min}}$ is the minimum value in the dataset of indicator j, and ${\text{x}}_{\text{j}}^{\text{max}}$ is the maximum value in the dataset of indicator j. The processed data ∈[0, 1].

2) Calculating the weight values. The weight values of each indicator need to be determined by comprehensively measuring the contrast and conflict. The conflict index between the j-th indicator and the others is $\sum_{\text{t}=\text{1}}^{\text{n}}\left(\text{1}-{\text{r}}_{\text{tj}}\right)$, where r_tj_ is the correlation coefficient between indicators t and j. If C_j_ represents the information contained in the j-th indicator, then C_j_ can be expressed as:

$$\begin{array}{c} C_{j}=\sigma_{j}\sum {\mathrm{\backslash nolimits}}_{t=\text{1}}^{n}\left(\text{1}-r_{tj}\right) \end{array}$$ (6)

The larger the C_j_, the more information the j-th indicator provides, and the higher the relative importance of the indicator. Therefore, the objective weight of the j-th indicator can be obtained as:


$$\beta_{j}=\frac{C_{j}}{\sum_{j=\text{1}}^{n}C_{j}}$$ (7)

The CRITIC method requires specific spatial data to be substituted for the calculation, so the results are not shown in this section, see Results for details.

#### 2.2.3 Composite weighting method AHP-CRITIC.

The weight values of the second-level indicators are calculated by AHP method and CRITIC method, with subjective weight α and objective weight β, respectively. To ensure the composite weight ω_i_ as close as possible to α_i_ and β_i_, without bias towards either side, the composite weight value is computed using the following formula:


$$\omega_{\text{i}}=\frac{\alpha_{\text{i}}\beta_{\text{i}}}{\sum \alpha_{\text{i}}\beta_{\text{i}}}$$ (8)

### 2.3 Data sources

The data used in this study are from the Ming’s postal system database built by the authors. Specifically, postal facility data were sourced from historical documents [39–41], followed by spatial positioning and calibration; spatial data was extracted from ASTER GDEM 30M digital elevation data [42] using ArcGIS spatial analysis tools. Major analysis tools include: Slope, used to generate slope raster data for each region; Focal Statistics, used to generate the geographical relief raster data for each region; Near Analysis, used to calculate the distance between the two nearest postal facilities; Thiessen Polygon, used to calculate the jurisdiction areas of Jidipu.

Statistical analysis was conducted with counties as the basic unit. For calculation convenience, except for the number of postal facilities, the average spatial values (elevation, slope, relief, distance, and jurisdiction area) of postal facilities within each county were taken as the corresponding values for the evaluation indicators. Notably, the acquired data exhibited inconsistent dimensions, precluding direct use in the evaluation system. To eliminate this impact, a score conversion system was employed to uniformly de-dimensionalize the data of each indicator, ensuring all indicators fell within the same order of magnitude for enhanced comparability.

## 3 Case analysis and results

### 3.1 Case selection

**Fig 4:**
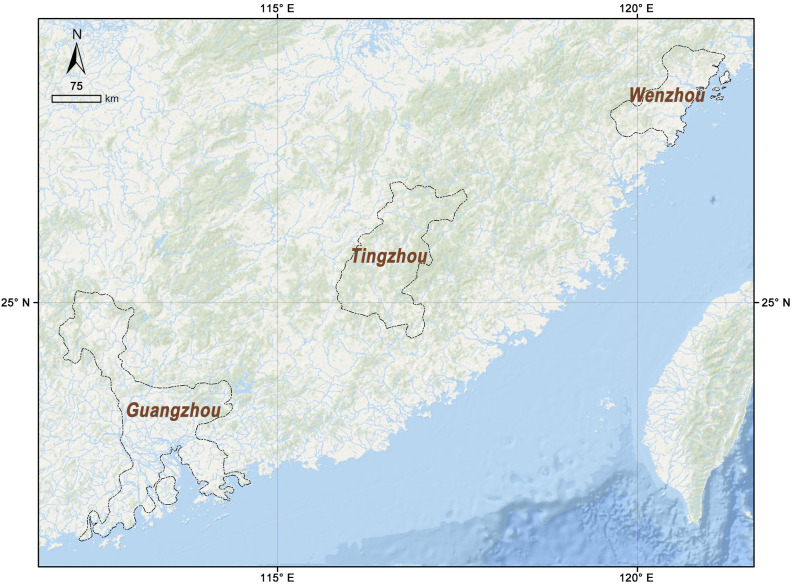
Location of the study cases: generated by the authors using ArcGIS Pro software.

Here, the coastal areas of the Ming Dynasty were taken as the study area. The Ming Dynasty’s territory featured a long, winding coastline, stretching from Liaodong in the north to Guangdong in the south, with Hainan Island situated offshore. From a military perspective, scholars typically divide the entire coastal area into seven defense zones, ordered from north to south: Liaodong, Bei Zhili (Northern Metropolitan Area), Shandong, Nan Zhili (Southern Metropolitan Area), Zhejiang, Fujian, and Guangdong [43]. Considering geographical location, functional attributes, topographical characteristics, and data availability, three representative regions were selected as study cases within the coastal area: Wenzhou (a typical region of coastal defense), Tingzhou (a typical region of transportation hub), and Guangzhou (a typical region of administrative center) (Fig 4).

#### 3.3.1 Wenzhou.

**Fig 5:**
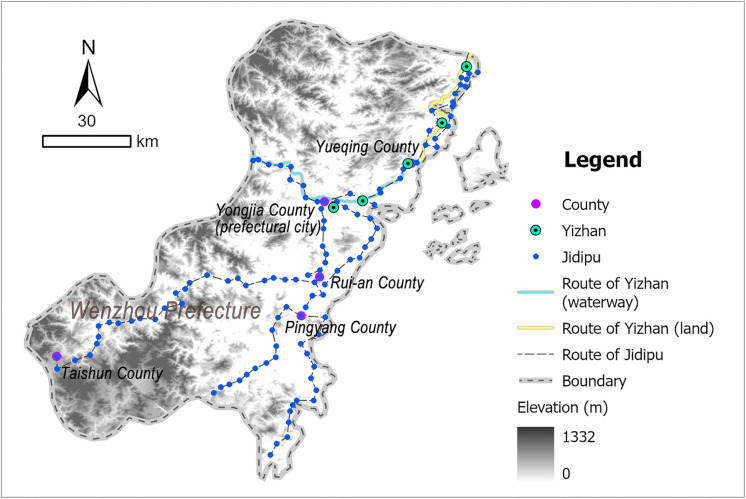
Distribution map of Wenzhou’s postal system: generated by the authors using ArcGIS Pro software.

Wenzhou was situated in southeastern Zhejiang Province, bordered by coastal waters to the east, mountainous terrain to the west, Taizhou Prefecture (within the same province) to the north, and Fujian Province to the south, numerous islands scattered along its coastline. During the Ming Dynasty, Japanese pirates frequently landed in Zhejiang via maritime routes influenced by monsoons and ocean currents, making Wenzhou a primary invasion target that suffered recurrent invasions. Its strategic position at the Zhejiang-Fujian border further underscored its critical role in safeguarding the security of both provinces [44]. As a result, the Ming government attached great importance to the construction of coastal defense in Wenzhou, establishing it as a representative case of coastal defense-oriented postal system development. According to the Ming-era *Wenzhou Prefecture Annals* (温州府志) [39], Wenzhou maintained 5 Yizhan and 115 Jidipu during this period (Fig 5 and S1 Table).

#### 3.3.2 Tingzhou.

**Fig 6:**
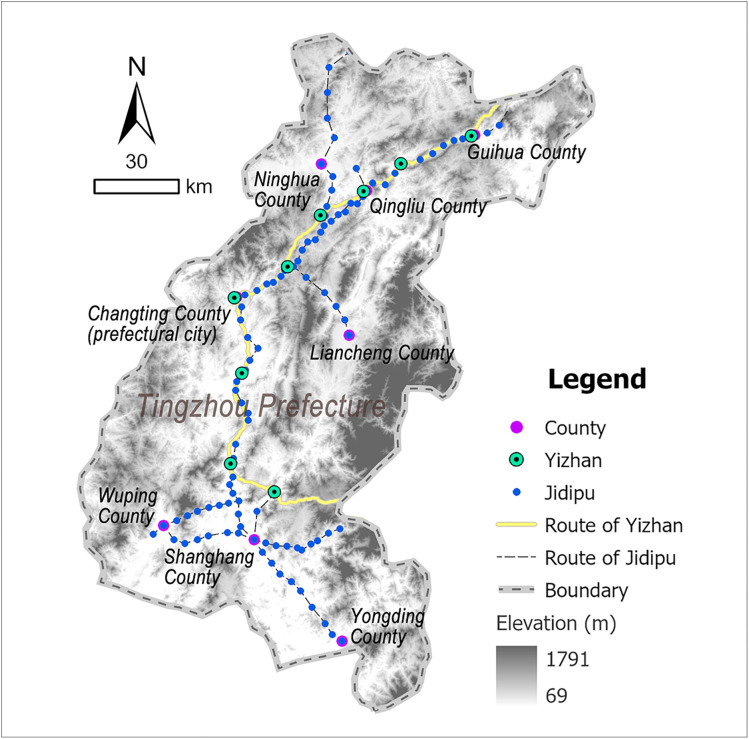
Distribution map of Tingzhou’s postal system: generated by the authors using ArcGIS Pro software.

Tingzhou Prefecture was situated in the southwestern Fujian Province, adjacent to Guangdong Province to the south and Jiangxi Province to the west, functioning as a transportation hub connecting the three provinces. Situated at the southern foothills of the Wuyi Mountains where the Ting River originates, Tingzhou featured elevated terrain characterized by deep ravines, overlapping mountain ridges, and fluvial barriers, forming a typical mountainous and hilly topography. Benefiting from its developed waterway networks, Tingzhou emerged as a critical nexus linking coastal and inland regions. Historically, goods from neighboring areas such as Guangdong and Jiangxi were first consolidated in Tingzhou, then distributed nationwide via its riverine transport systems. This strategic positioning established Tingzhou as a representative example of inland strategic hubs within coastal provinces [45]. According to the Ming-era annals of *Comprehensive Gazetteer of Fujian* (八闽通志) [40], Tingzhou operated 9 Yizhan and 104 Jidipu during this period (Fig 6 and S2 Table).

#### 3.3.3 Guangzhou.

**Fig 7:**
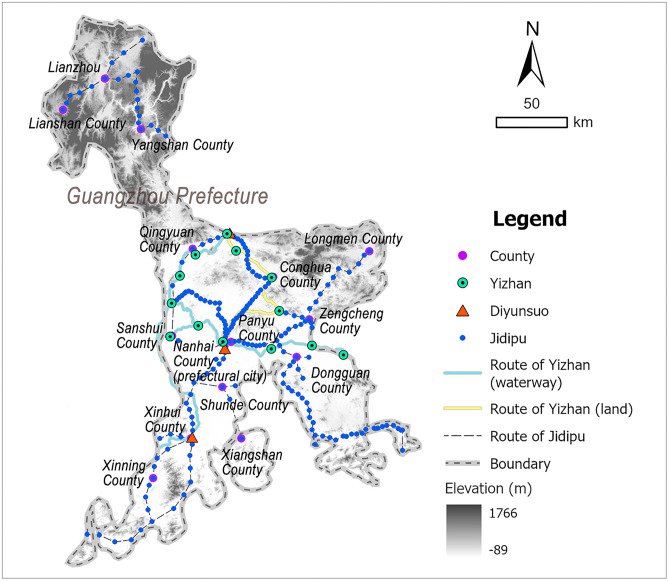
Distribution map of Guangzhou’s postal system: generated by the authors using ArcGIS Pro software.

Guangzhou Prefecture was the seat of the provincial government, located in the central Guangdong Province, bordering Huguang Province in the north, Guangxi Province in the northwest, and the sea in the south, spanning a vast geographic range from north to south. The terrain within Guangzhou was flat and low-lying: its southern coastal area featured a concave bay, while the central region comprised a deltaic plain formed by the confluence of three rivers. During the Ming Dynasty, Guangzhou served as the political, economic, and cultural centers of Guangdong. As an international trade port, its exceptional economic prosperity frequently attracted covetous attention from Japanese pirates and maritime bandits, thereby constituting a strategically critical coastal defense frontier [46]. According to the Ming-era *Guangdong Province Annals* (广东通志) [41], the postal system in Guangzhou consisted of 14 Yizhan, among which Huaiyuan Yi was exclusively for the reception of envoys and thus excluded from accessibility analysis, and a total of 13 Yizhan, 3 Diyunsuo, and 202 Jidipu were included in the study (Fig 7 and S3 Table).

### 3.2 Weights calculation

Subjective weights for the postal system’s accessibility were calculated using the AHP method, with the detailed process provided in S1 Appendix. The calculation results are as follows:

First-level weights (corresponding to the three postal facility types: Yizhan, Diyunsuo, Jidipu): (0.30, 0.16, 0.54).

Second-level weights for Yizhan, Diyunsuo and Jidipu: (0.33, 0.14, 0.14, 0.14, 0.25), (0.20, 0.20, 0.20, 0.40) and (0.17, 0.11, 0.11, 0.11, 0.25, 0.25).

All weight values passed the consistency test, confirming their validity.

Objective weights for the postal system’s accessibility are calculated using the CRITIC method, with results as follows:

Second-level weights for Yizhan, Diyunsuo and Jidipu: (0.24, 0.23, 0.17, 0.13, 0.23), (0.16, 0.13, 0.18, 0.53) and (0.24, 0.20, 0.13, 0.14, 0.15, 0.14).

**Fig 8:**
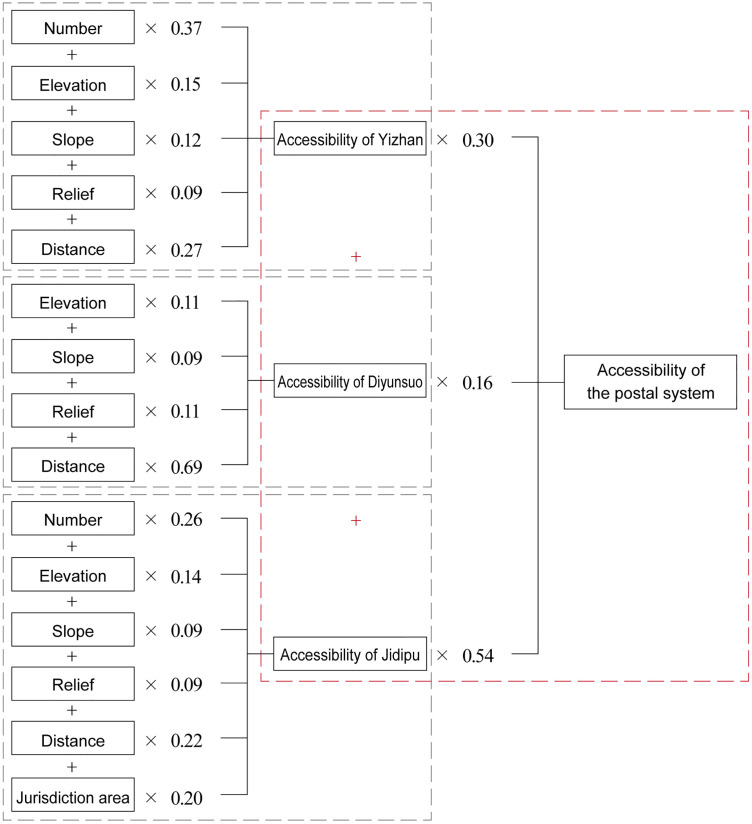
The accessibility evaluation model for the postal system in the Ming Dynasty.

By substituting the subjective and objective weights into Eq. (8) for computation, the final accessibility evaluation model for the Ming postal system was derived, as illustrated in Fig 8.

### 3.3 Accessibility in Wenzhou

**Fig 9:**
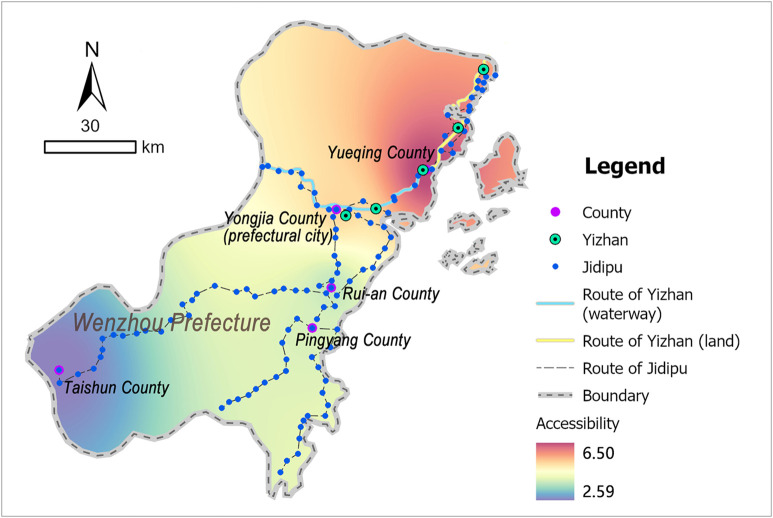
Accessibility distribution map of the postal system in Wenzhou: generated by the authors using ArcGIS Pro software.

The dimensionalized data of the postal system in each county are presented in S2 Appendix. Substituting them into the evaluation model for calculation, the final analysis results were obtained, as shown in Table 3. To visually illustrate the spatial relationship between postal system accessibility and geographic location, the Inverse Distance Weighting tool in the ArcGIS platform was used to project the final scores onto the postal system distribution map. As shown in Fig 9, colors closer to red indicate higher accessibility, while colors closer to blue indicate lower accessibility.

**Table 3:** Accessibility scores of the postal system in Wenzhou.

Prefecture	County	Accessibility score of the postal system
Wenzhou	Yongjia	5.85
	Yueqing	6.50
	Rui’an	3.88
	Pingyang	3.87
	Taishun	2.59

In the accessibility analysis results of Wenzhou’s postal system, Yueqing County (6.50) and Yongjia County (5.85) in the northern region formed the high-value core zones, followed by Pingyang (3.88) and Rui’an (3.87) counties, while Taishun County (2.59) in the southern inland area ranked as the lowest-value zone across the entire prefecture. This presents an overall spatial pattern characterized by high accessibility in the northern coastal areas and low accessibility in inland area (Fig 9).

### 3.4 Accessibility in Tingzhou

**Fig 10:**
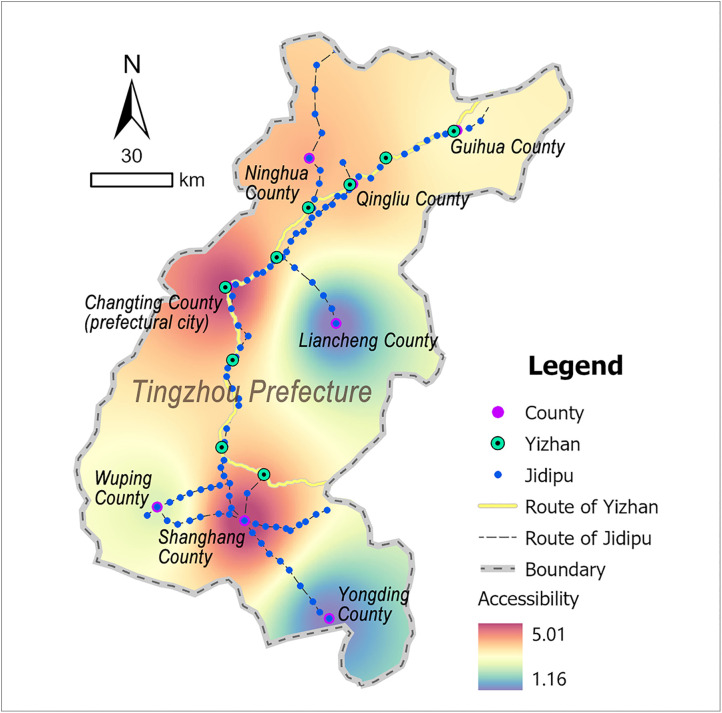
Accessibility distribution map of the postal system in Tingzhou: generated by the authors using ArcGIS Pro software.

The calculation results of the postal system in Tingzhou are shown in Table 4. Its accessibility differentiation of Tingzhou’s postal system exhibited a pronounced north-south axial dominance spatially (Fig 10): Constrained by the its elongated north-south geographical form and restricted east-west space, the postal the postal system was distributed longitudinally along the north-south axis, forming a gradient pattern with Shanghang County (5.01) and Changting County (4.63) as highest values.

**Table 4:** Accessibility scores of the postal system in Tingzhou.

Prefecture	County	Accessibility score of the postal system
Tingzhou	Changting	4.63
	Ninghua	3.90
	Shanghang	5.01
	Wuping	2.37
	Qingliu	3.85
	Liancheng	1.16
	Guihua	3.34
	Yongding	1.45

### 3.5 Accessibility in Guangzhou

**Fig 11:**
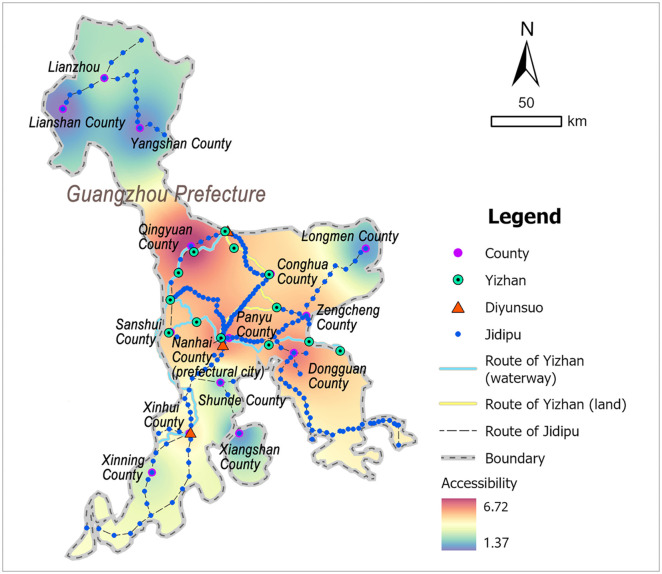
Accessibility distribution map of the postal system in Guangzhou: generated by the authors using ArcGIS Pro software.

The calculation results of the postal system in Guangzhou are shown in Table 5. Guangzhou administered numerous counties, necessitating grouped analysis for systematic discussion. High-accessibility areas (>5) concentrated in central and southeastern regions, including 7 counties such as Qingyuan (6.72), Nanhai (6.59), Dongguan (6.20), and Panyu (5.84). Moderate-accessibility areas (2–5) were predominantly distributed along the southwestern coast, namely Xinhui County (4.12), Xinning County (2.96), and Shunde County (2.42), as well as Longmen County (2.07) at the central periphery. Low-accessibility areas (<2) concentrated in northern territories such as Lianzhou (1.65) and Lianshan (1.37). Spatial analysis revealed complex accessibility patterns in Guangdong, exhibiting a general characteristics of high in the central and southeastern areas, middle in the southwestern area, and lowest in the northern area (Fig 11).

**Table 5:** Accessibility scores of the postal system in Guangzhou.

Prefecture	County	Accessibility score of the postal system
Guangzhou	Nanhai	6.59
	Panyu	5.84
	Shunde	2.42
	Dongguan	6.20
	Sanshui	5.43
	Conghua	5.31
	Xinhui	4.12
	Xinning	2.96
	Xiangshan	1.93
	Zengcheng	5.52
	Qingyuan	6.72
	Longmen	2.07
	Lianzhou	1.65
	Yangshan	1.67
	Lianshan	1.37

## 4 Discussion

### 4.1 Accessibility characteristics and regional layout priorities

This spatial differentiation of Wenzhou’s accessibility originated from the synergistic effects of military defense and political demands. Northern Yueqing County, bordering the sea to the east and Taizhou Prefecture to the north, served as both the land gateway for the provincial official route into Wenzhou and the front-line of coastal defense, which required a robust postal system to ensure unobstructed information flow and sufficient material supplies. In the 23rd year of the Hongwu era (1390), the Ming government specifically established a coastal postal route in Yueqing for transmitting military information, making the number of Yizhan and Jidipu the highest in Wenzhou [47]. As a result, the accessibility of Yueqing’s postal system was the highest among all counties. As the seat of Wenzhou Prefecture, Yongjia County functioned as both the political center and regional postal hub. Its accessibility had to remain high to enable administrators to process and gain control of information across the entire prefecture efficiently. Though southern Pingyang and Rui’an were coastal counties, their location in inland bays reduced Japanese pirate threats, leading to a less developed postal system. The inland Taishun County, situated in mountainous terrain, saw its postal routes wind through narrow valleys due to topographic constraints. The rugged terrain limited the construction of postal facilities, coupled with minimal military conflicts, leading to only sparse Jidipu to meet basic information transmission needs. Consequently, the accessibility of Taishun County’s postal system remained significantly lower than other counties. Essentially, such spatial difference was evidence of the Ming Dynasty postal system’s “coastal defense prioritization and terrain-adaptive configuration”. Geographical conditions determine the foundation of resource allocation, while military and political strategies dominate the prioritization of resource distribution; together, these two factors shape the unbalanced pattern of the postal route network.

Tingzhou’s spatial configuration was fundamentally shaped by terrain features and transportation demands: Shanghang County, located in the low-altitude valley of the lower Ting River, served as a tri-provincial transportation hub at the Fujian-Guangdong-Jiangxi border. As the only land link to Zhangzhou Prefecture and a critical channel for the Ting River waterway, its frequent cross-regional logistics required dense deployment of postal facilities. Changting County, the prefectural seat, leveraged its central location to enhance postal deployment, with its postal system maintaining high accessibility to meet the rigid demand for official document transmission. Ninghua and Qingliu counties, which guarded the official route to Shaowu Prefecture, sustained moderate accessibility through transit traffic. By contrast, the off-axis counties (Guihua, Wuping, Yongding, and Liancheng), constrained by the terrain of the Wuyi Mountains, not only lacked inter-prefectural postal route connections but also faced difficulties in developing branch routes, reducing most of their postal stations to marginal nodes. The spatial differentiation in Tingzhou’s postal accessibility fundamentally arose from the interplay of geographical constraints and political imperatives. Given terrain’s significant impact on transmission, postal facilities in mountainous areas were prioritized in lower-elevation, relatively flat zones; this concentration of infrastructure, coupled with unimpeded route connectivity, resulted in optimal accessibility. Meanwhile, the prefectural political center, which controlled the entire prefecture’s postal system, naturally maintained elevated accessibility levels.

The accessibility pattern of Guangzhou’s postal system exhibited the highest complexity, driven by multifactorial influences: Qingyuan County in the north-central region functioned as a transportation hub via the North River waterway, directly connecting to the prefectural city and enabling efficient material distribution. Nanhai and Panyu Counties served as the political and economic center of both Guangzhou and the entire province, as well as a foreign trade hub in the Ming Dynasty. Envoys from Champa, Siam, and other southern neighboring regions conducted business here [2], necessitating sufficient postal facilities to ensure smooth cargo transportation. Consequently, the postal system in this central area achieved the highest accessibility. Coastal accessibility variations mirrored defense strategies: Southeast Dongguan County, governing the east coast of Guangzhou Bay with an extensive coastline, served as the coastal defense frontline guarding the mainland entrance. Its strategic role in protecting the estuary and urban security drove high demand for postal facilities, corresponding to enhanced postal accessibility. By contrast, southwestern coastal counties (Xinhui and Xinning) exhibited lower accessibility than Dongguan. This disparity arose because the southwest flank of Guangzhou Bay was shielded by Xiangshan Island, forming a priority defense line for Xinhui and Xinning and reducing Japanese pirate incursions in the region. As a result, southern coastal counties displayed a spatially diminishing accessibility gradient from east to west. Northern Lianzhou, far from the political core and coastal defense frontline, had minimal transmission demand, with only a small number of postal facilities built, leading to low accessibility. Generally, the areas with high postal accessibility in Guangzhou exhibit three types of characteristics: transportation hub to the north of the prefectural city, the seat of the prefectural and provincial government, and crucial coastal defense area. The regional spatial distribution of Guangzhou’s postal system was not only in line with its the special positioning of political and economic center, but also closely related to military defense, as well as having a certain correlation with the geographic environment.

In summary, the regional layout of the Ming postal system in coastal areas prioritized the coast and administrative centers, with inland areas as secondary priorities, while in local regions, emphasis was placed differently according to the regional strategic importance and transmission demands. There were three key regions in the distribution of postal system: First, coastal prefectures and counties, which were important components of the coastal defense system, exhibiting the highest accessibility to ensure logistical support for coastal defense. Second, prefectural administrative centers, which were functioned as political-economic centers as well as operation hubs of the postal system. The efficient postal system ensured the rapid control of information across the whole territory, eliminating the barriers between the central and remote areas. Third, areas along the main transportation arteries, which undertook the transmission between provincial centers and prefectures. Other regions, restricted by factors such as geographical conditions, generally had relatively low postal accessibility

### 4.2 Influencing factors within historical context

The paramount driving force behind this development was military necessity during the Ming Dynasty’s turbulent founding period and subsequent regime consolidation. In 1368, Emperor Zhu Yuanzhang established the Ming Dynasty, but the newly formed Ming regime faced instability amid severe internal and external challenges. Externally, persistent coastal raids by Japanese pirates colluding with reactionary forces exerted immense pressure on southeastern coastal defenses. Internally, prolonged warfare had devastated the economy and fueled social unrest, compelling the new regime to centralize control and fortify its authority [48]. Thus, Emperor Zhu not only prioritized the development of military forces but also implemented measures to strengthen feudal centralization. Given that “post was the bloodline of the world”, restoring the transmission system became a priority. Against this backdrop, the Ming Dynasty’s postal system was militarized from the outset of its restoration [2]. Imperial decrees explicitly stipulated that the core tasks of Yizhan, Diyunsuo and Jidipu were “escorting envoys, transmitting military information and delivering military supplies” [49]. In coastal areas where military defense was crucial, the postal system exhibited particularly strong militarization and became an integral part of the coastal defense system, which served the defensive needs in terms of the layout, regulation, organization and management. As a result, the postal system was more densely distributed in areas with higher defense priorities.

However, socioeconomic transformation in the mid-Ming period emerged as a key driver of functional expansion and institutional evolution within the postal system.With the stabilization of imperial rule and gradual economic development, military-focused postal infrastructure fell short of proliferating societal demands. Heightened population mobility, the expansion of long-distance trade and increasingly complex local administration collectively intensified needs for efficient information exchange and small-scale logistics. Consequently, starting from the mid-Ming era, the postal system underwent significant adaptive changes: institutional restrictions that limited postal services exclusively to military dispatches were abolished. Beyond military matters, ordinary documents and goods could also be transmitted by post, which gradually extended its reach into national politics, military, economy, transportation and culture [49]. The prefectures where the administrative centers were located, as a gathering place for local information and supplies, naturally became the key nodes of the postal system. As postal regulations broke free from the “military intelligence only” constraint and permitted the transmission of ordinary documents and goods, the network evolved from a military artery into an economic link. Postal facilities in administrative centers and transportation hubs thus transformed into distribution nodes for local materials and information. This institutional shift reflected the system’s responsiveness to socioeconomic development, solidifying its role as a multifunctional nexus that integrated regional goods and information distribution alongside its existing military functions.

Notably, the postal system’s spatial configuration and operational patterns remained fundamentally constrained by geographical conditions and technological limitations. “Adapting to local conditions” became an indispensable empirical principle for its construction and management. In topographically challenging regions, where the original layout logic (e.g., placing Yizhan at 60 Ming li intervals and Jidipu every 10 Ming li, where 1 Ming li equals 0.55 km) [50] proved largely impracticable, actual delivery needs were prioritized to accommodate the environment. On this condition, postal facilities were preferentially built at the busiest transportation hubs, while also being strategically situated on relatively flat terrain to meet regional communication and transportation needs.

In summary, the postal system of the Ming Dynasty emerged as a historical product shaped by multiple sociopolitical forces, including state centralization, military strategy, geographical constraints, and socioeconomic dynamics. To understand its accessibility, contextualization within this complex framework is essential. Only then can its planning logic and operational efficacy be fully comprehended. Being the “bloodline of the empire” [2], the postal system determined the transmission of governmental orders and frontier emergency information, with its accessibility closely tied to the rise and fall of the Ming Dynasty. Consequently, systematic planning of the postal system was imperative to ensure its efficient operation amid limited resources.

### 4.3 Modern continuity of the ancient postal system

Contextualized within broader historical frameworks, the postal system functioned not only as an instrument of authoritarian governance but also progressively catalyzed the expansion of trade networks over time. Through its nationwide transmission infrastructure, this system provided foundational support for cross-regional commerce. It accelerated interregional flows of personnel and goods, with its route planning establishing stable trade corridors (e.g., the Silk Road) that reduced transportation costs and risks. Simultaneously, the transmission of administrative decrees, market intelligence, and commercial instruments enhanced transactional efficiency and trustworthiness.

Examining contemporary global and national development strategies, the spatial legacy of ancient postal systems reveals remarkable continuity and relevance. From ancient postal system and the Silk Road to the contemporary national strategy, transportation networks have persistently driven domestic-international connectivity. The postal system significantly facilitated cross-regional civilizational trade, making ancient China the Eurasian trade nexus while driving the prosperity of cities along the route and the integration of diverse cultures. The Belt and Road Initiative inherits this historical lineage, reconstructing Afro-Eurasian connectivity through modern “neo-postal system”: ports, railways, and digital infrastructure. At the regional level, the Guangdong-Hong Kong-Macao Greater Bay Area development mirrors postal system principles by emphasizing both infrastructure construction and institutional rules, echoing historical practices where standardized management enhanced cross-jurisdictional efficiency. Its planning of creating one-hour living circle and efficient logistics networks manifests the nodal radiation concept of ancient postal system, with core hubs (ports, airports, high-speed rail stations) serving modern city clusters.

In general, the postal system represent not only an early paradigm of logistics and information management that laid the groundwork for modern trade infrastructure, but also embedded core principles into subsequent developmental practices, such as strategic nodal deployment, standardized protocols and cross-regional coordination. These concepts transcend time and space, continuously evolving within contemporary global systems: from international trade frameworks like the Belt and Road Initiative and regional collaboration models such as the Guangdong-Hong Kong-Macao Greater Bay Area, to digital delivery networks and telecommunication systems. They form an unbroken historical thread linking ancient trade arteries to modern global supply chains and regional synergy networks.

### 4.4 Exploration of heritage conservation strategies

The regional layout characteristics of the Ming postal system can provide historically contextualized references for contemporary heritage protection and spatial planning. In heritage protection, it is suggested to establish a hierarchical conservation framework rely on the integration of “defense-politics-transportation” structure. For postal heritage in coastal areas, their military defense attributes must be prioritized by incorporating them into a composite military heritage conservation system alongside Ming military fortresses and beacon towers. Strengthening its military memory through the construction of site parks and the restoration of emergency delivery scenes. Regarding postal heritage in administrative centers, conservation efforts should emphasize their spatial connectivity with historical urban axes, preserving their spatial characteristics as regional transportation hubs. Meanwhile, their functional role in historical governance should be interpreted through exhibitions and educational programs. For postal heritage along the major traffic arteries, attention should be paid to the spatial continuity of the network, avoiding fragmentation by modern urbanization. GIS technology can be used to accurately compute the postal routes, enabling the demarcation of protection corridors in urban planning to maintain heritage connectivity.

In spatial planning, the planning wisdom of the Ming postal system can be drawn on to achieve the organic integration of heritage and contemporary spatial structure. Urban renewal initiatives should prioritize preserving the pattern of historical streets where postal facilities are located. Combined with the functional needs of mordern cities, postal sites can be transformed into regional transportation points, cultural service centers, etc., so as to realize the functional conversion from “historical hubs” to “modern nodes”. For regional transportation planning, reference can be made to the hierarchical structure of arteries and branch lines of the Ming postal system: the accessibility of remote areas can be improved by optimizing branch line connections. Meanwhile, postal heritage can be incorporated into the planning of greenways and slow-traffic systems, embedding historical transportation into the fabric of modern urban development.

### 4.5 Research applicability and limitations

The AHP-CRITIC composite weighting method effectively balances subjective and objective considerations, demonstrating good suitability for evaluating accessibility in ancient postal system. Critically, the choice of objective weighting method significantly impacts model outcomes. Therefore, correlation analysis and multiple weighting approaches were adopted to validate methodological appropriateness.

Specifically, taking the five sub-indicators of Jidipu as an example, the Spearman method [51] was adopted for indicator correlation analysis firstly. The results (Table 6) revealed significant correlations among elevation, slope, and relief degree, as well as among number, distance, and jurisdiction area, aligning with the characteristic of CRITIC method. To further verify this conclusion, the entropy, CV, and CRITIC methods were respectively employed to compute the indicator weights (Table 7). In the entropy and CV methods, distance and jurisdiction area accounted for extremely high weights respectively; whereas CRITIC effectively neutralized this weight deviations. Dual analysis confirmed CRITIC as the ideal objective weighting method for accessibility assessment in ancient postal system.

**Table 6:** Correlation analysis results of indicators.

	Number	Elevation	Slope	Relief	Distance	Jurisdiction area
**Number**	1 (0.000***)	−0.145 (0.461)	−0.013 (0.947)	0.036 (0.855)	−0.576 (0.001***)	−0.828 (0.000***)
**Elevation**	−0.145 (0.461)	1 (0.000***)	0.845 (0.000***)	0.862 (0.000***)	0.085 (0.668)	0.363 (0.058*)
**Slope**	−0.013 (0.947)	0.845 (0.000***)	1 (0.000***)	0.967 (0.000***)	−0.041 (0.838)	0.241 (0.217)
**Relief**	0.036 (0.855)	0.862 (0.000***)	0.967 (0.000***)	1 (0.000***)	−0.03 (0.881)	0.195 (0.319)
**Distance**	−0.576 (0.001***)	0.085 (0.668)	−0.041 (0.838)	−0.03 (0.881)	1 (0.000***)	0.747 (0.000***)
**Jurisdiction area**	−0.828 (0.000***)	0.363 (0.058*)	0.241 (0.217)	0.195 (0.319)	0.747 (0.000***)	1 (0.000***)

Note: Figures in brackets represent significance levels, where ***, **, and * denote significance at the 1%, 5%, and 10% levels, respectively.

**Table 7:** Comparison of three objective weighting methods.

Indicator	Weights by entropy method	Weights by CV method	Weights by CRITIC method
Number	0.1103	0.1250	0.2444
Elevation	0.2327	0.1785	0.2030
Slope	0.0694	0.0969	0.1302
Relief	0.0689	0.0961	0.1390
Distance	0.2928	0.2854	0.1466
Jurisdiction area	0.2259	0.2181	0.1367

Significantly, the evaluation framework synthesized accessibility context with the operational characteristics of ancient postal system, ensuring its applicability extends beyond specific cases. This is because the objective weight method computes weights based on specific data, incorporating topographic conditions and postal system distribution patterns. This enables the accessibility evaluation framework to be widely applicable to diverse scenarios across ancient postal networks, providing a reliable and universal analytical tool for related research.

However, the limitation of this study still exists. Due to the extensive postal system of the Ming Dynasty, it required mass of efforts to locate each postal facility by deeply analyzing historical documents from different periods and repeatedly comparing them for accuracy. Therefore, it is difficult to comprehensively sort out the postal system along the entire coastal line in one study, and representative case studies are needed. In subsequent research, the spatial scale of the study will gradually be expanded. First, the postal system in the northern coastal area should be sorted out and systematically analyzed, exploring the spatial layout differences and commonalities in the north and south, and summarizing the overall spatial rules of ancient Chinese postal system in the coastal area. Next, the study scope will be further extended to the areas along the Great Wall to explore how inland defense shaped postal system, and conduct cross-geomorphic comparison with coastal areas. Methodologically, the established evaluation framework will be utilized, employing the CRITIC method to compute differentiated weights based on regional topographic conditions and distribution patterns of the postal system, ensuring effective adaptation to accessibility evaluation across diverse geomorphic contexts. As research advances, efforts are also dedicated to collecting additional historical materials, exploring more scientific data processing methods, and enhancing the accuracy of the postal system database, so as to overcome the limitations of the existing materials and methods, promoting further research on ancient postal system.

## 5 Conclusion

In this study, the concept of accessibility was introduced, and a hierarchical evaluation model applicable to postal system was constructed based on the composite weighting method AHP-CRITIC. The model comprehensively assesses three dimensions and four factors, conducting quantitative analysis of three typical cases in the coastal area. The results demonstrated distinct accessibility patterns across different regions:

1) Wenzhou exhibited a spatial pattern of high in the northern and coastal areas, low in the inland area. Specifically, Yueqing County showed the highest accessibility, followed by Yongjia, Pingyang, and Rui’an counties, while Taishun County exhibited the lowest accessibility. This spatial hierarchy primarily reflected maritime defense strategic deployments.
2) Tingzhou demonstrated north-south axial dominance. Counties along the longitudinal corridor—Shanghang, Changting, Ninghua, and Qingliu—maintained higher accessibility, whereas off-axis counties like Wuping, Liancheng, and Guihua showed reduced performance. This configuration correlated with topographic constraints and administrative priorities.
3) Guangzhou presented complex accessibility characteristics: central and southeastern regions (Qingyuan County, Nanhai County, Panyu County, Dongguan County, etc.) achieved high accessibility, southwestern areas (Xinhui County, Xinning County, and Shunde County) showed moderate levels, and northern territories of Lianzhou ranked lowest. This spatial configuration aligned with its political-economic centrality, coastal defense demands, and geographical conditions.

Further, three representative coastal areas were quantitatively analyzed, from which the regional layout characteristics of the postal system in the Ming Dynasty were summarized. The postal system along the coast had the most distinctive attribute of satisfying coastal defense needs; the postal system near the inland area was more affected by geographical conditions; the postal system in the political center had to serve political and economic needs as well as coastal defense goals. Consequently, three critical areas for postal system planning were identified: the coastal areas, the administrative centers, and the key transportation arteries. Analysis revealed that this regional spatial characteristics was shaped by multiple sociopolitical forces, including state centralization, military strategy, geographical constraints, and socioeconomic dynamics.

## Supporting information

S1 Appendix Detailed process of AHP method. (DOCX)

S2 Appendix Detailed steps of data processing. (DOCX)

S1 Table Details of Wenzhou’s postal system in the Ming Dynasty. (PDF)

S2 Table Details of Tingzhou’s postal system in the Ming Dynasty. (PDF)

S3 Table Details of Guangzhou’s postal system in the Ming Dynasty. (PDF)
