# Supplementary material for: Exploring the regional layout characteristics of ancient Chinese postal system in coastal areas based on AHP-CRITIC evaluation approach
Source: PLoS One. 2025 Sep 25;20(9):e0333348. doi: 10.1371/journal.pone.0333348 (PMC12463204; doi:10.1371/journal.pone.0333348)
Supplement: S1 Appendix — (DOCX) [file pone.0333348.s001.docx]

**S1 Appendix** Detailed process of AHP method

**1. Constructing the judgment matrix**

To calculate the weights using the AHP method, it is first necessary to access the importance degree of each evaluation indicator. By pairwise comparing the importance degree of indicators in the same set relative to the corresponding indicators at the upper level, and using the numbers 1-9 and their reciprocal as standardized metrics (Table S1-1), the judgment matrix is constructed.

**Table S1-1 Definitions of the metrics**

| Intensity of importance | Definition |
| --- | --- |
| 1 | The former and the latter are equally important |
| 3 | The former is slightly more important than the latter |
| 5 | The former is obviously more important than the latter |
| 7 | The former is strongly more important than the latter |
| 9 | The former is extremely more important than the latter |
| 2，4，6，8 | The intermediate value of the above adjacent judgments |
| Reciprocals | If activity i has one of the above nonzero numbers assigned to it when compared with activity j, then j has the reciprocal value when compared with i |

The comparison of the importance degree is dependent on the properties of the postal system and the the degree to which the indicator itself affects accessibility. Taking the indicators at the first level as an example, Yizhan served as the transmission facility connecting prefectures, Diyunsuo was mainly responsible for the delivery of goods between provincial centers, and Jidipu undertook most of the transmission affairs within the county. Therefore, for the postal system of a county, Jidipu was inevitably the most important, followed by Yizhan, and finally Diyunsuo. According to the order of importance, a judgment matrix for the first level can be constructed, as shown in Table S1-2. Likewise, based on the analysis of each indicator, combined with the concept of accessibility and the comprehensive evaluation of influencing factors, judgment matrices for the remaining groups were constructed through pairwise comparisons, as shown in Tables S1-3, S1-4, and S1-5.

**Table S1-2 Judgment matrix for the first level of the postal system’s accessibility evaluation system**

|  | **Accessibility of Yizhan** | **Accessibility of Diyunsuo** | **Accessibility of Jidipu** |
| --- | --- | --- | --- |
| **Accessibility of Yizhan** | 1 | 2 | 1/2 |
| **Accessibility of Diyunsuo** | 1/2 | 1 | 1/3 |
| **Accessibility of Jidipu** | 2 | 3 | 1 |

**Table S1-3 Judgment matrix for the accessibility of Yizhan**

|  | **Number** | **Elevation** | **Slope** | **Relief** | **Distance** |
| --- | --- | --- | --- | --- | --- |
| **Number** | 1 | 2 | 2 | 2 | 2 |
| **Elevation** | 1/2 | 1 | 1 | 1 | 1/2 |
| **Slope** | 1/2 | 1 | 1 | 1 | 1/2 |
| **Relief** | 1/2 | 1 | 1 | 1 | 1/2 |
| **Distance** | 1/2 | 2 | 2 | 2 | 1 |

**Table S1-4 Judgment matrix for the accessibility of Diyunsuo**

|  | **Elevation** | **Slope** | **Relief** | **Distance** |
| --- | --- | --- | --- | --- |
| **Elevation** | 1 | 1 | 1 | 1/2 |
| **Slope** | 1 | 1 | 1 | 1/2 |
| **Relief** | 1 | 1 | 1 | 1/2 |
| **Distance** | 2 | 2 | 2 | 1 |

**Table S1-5 Judgment matrix for the accessibility of Jidipu**

|  | **Number** | **Elevation** | **Slope** | **Relief** | **Distance** | **Jurisdiction area** |
| --- | --- | --- | --- | --- | --- | --- |
| **Number** | 1 | 2 | 2 | 2 | 1/2 | 1/2 |
| **Elevation** | 1/2 | 1 | 1 | 1 | 1/2 | 1/2 |
| **Slope** | 1/2 | 1 | 1 | 1 | 1/2 | 1/2 |
| **Relief** | 1/2 | 1 | 1 | 1 | 1/2 | 1/2 |
| **Distance** | 2 | 2 | 2 | 2 | 1 | 1 |
| **Jurisdiction area** | 2 | 2 | 2 | 2 | 2 | 1 |

**2. Calculating the weight values**

Taking the judgment matrix for the first level as an example, the calculation procedures are as follows:

$$A=\left| \begin{matrix} 1 & 2 & \frac{1}{2} \\ \frac{1}{2} & 1 & \frac{1}{3} \\ 2 & 3 & 1 \end{matrix} \right|$$

① Calculate the product of each row of numbers in the judgment matrix A, and get Mi.

② Calculate the cubic root of Mi, and get .

③ Normalize $\overline{\alpha_{\text{i}}}=\text{（}\overline{\alpha_{1}},\overline{\alpha_{2}},\overline{\alpha_{3}})$ by using the following equation.

$\alpha_{i}=\frac{\bar{\alpha_{i}}}{\sum_{i=1}^{3} \bar{\alpha_{i}}}$ (1)

Then, $\overline{\alpha_{\text{i}}}=\text{（}\overline{\alpha_{1}},\overline{\alpha_{2}},\overline{\alpha_{3}})$is the weight value of judgment matrix A.

According to the above steps, the weight value of each judgment matrix can be obtained. The weight values of the indicators for the postal system’s accessibility are (0.2970, 0.1634, 0.5396), the weight values of the indicators for Yizhan’s accessibility are (0.3264, 0.1421, 0.1421, 0.1421, 0.2473), the weight values of the indicators for Diyunsuo’s accessibility are (0.2, 0.2, 0.2, 0.4), and the weight values of the indicators for Jidipu’s accessibility are (0.1749, 0.1102, 0.1102, 0.1102, 0.2473, 0.2473).

**3. Consistency test**

As for whether the weight values above are reasonable or not, the consistency test of the judgment matrix is needed. The test equation is as follows:

$CR=\frac{\mathrm{CI}}{\mathrm{RI}}$ (2)

where CR is the random consistency ratio of the matrix, CI is the general consistency index of the matrix, and RI is the average random index of the judgment matrix which depends on n shown in Table S1-6. The calculation equation of CI is given as follows:

$CI=\frac{\lambda_{\max}-n}{n-1}$ (3)

$\lambda_{\max}=\sum_{i=i}^{n} \frac{{(A\alpha)}_{i}}{n\alpha_{i}}$ (4)

where λmax is the maximum eigenvalue of the matrix.

**Table S1-6 RI values**

| **n** | 1 | 2 | 3 | 4 | 5 | 6 | 7 | 8 | 9 |
| --- | --- | --- | --- | --- | --- | --- | --- | --- | --- |
| **RI** | 0 | 0 | 0.58 | 0.90 | 1.12 | 1.24 | 1.32 | 1.41 | 1.45 |

If CR is less than 0.1, or if λmax =n and CI=0, then the matrix can be considered as having an acceptable consistency, and the weight values take effect. Otherwise, the judgment scales in matrix A should be reviewed.

According to the above procedures, calculation results are shown as follows. In the matrix for the postal system’s accessibility, CR=0.0088＜0.1; in the matrix for Yizhan’s accessibility, CR=0.0132＜0.1; in the matrix for Diyunsuo’s accessibility, λmax=4, CI=0; and in the matrix for Jidipu’s accessibility, CR=0.013＜0.1. The calculation results show that the judgment matrices listed have acceptable consistency and the weight values are effective.
