# Supplementary material for: Exploring the regional layout characteristics of ancient Chinese postal system in coastal areas based on AHP-CRITIC evaluation approach
Source: PLoS One. 2025 Sep 25;20(9):e0333348. doi: 10.1371/journal.pone.0333348 (PMC12463204; doi:10.1371/journal.pone.0333348)
Supplement: S2 Appendix — (DOCX) [file pone.0333348.s002.docx]

**S2 Appendix** Detailed steps of data processing

**1. Data statistics**

After obtaining the relevant spatial data of the postal system in Wenzhou, Tingzhou, and Guangzhou, the data was averaged on a county as the corresponding values for the evaluation indicators of the county, facilitating further calculations. The statistical results are shown in Tables S2-1, S2-2 and S2-3. The statistical results are shown in Tables S2-1, S2-2 and S2-3.

**Table S2-1 Spatial data statistics of Yizhan in Wenzhou, Tingzhou and Guangzhou**

| **Prefecture** | **County** | **Number** | **Average elevation**  **（m）** | **Average slope**  **（°）** | **Average relief**  **（m）** | **Average distance**  **（km）** |
| --- | --- | --- | --- | --- | --- | --- |
| Wenzhou | Yongjia | 1 | 10 | 5.05 | 18 | 10.21 |
|  | Yueqing | 4 | 20 | 8.35 | 32.25 | 16.85 |
| Tingzhou | Changting | 3 | 360.67 | 8.97 | 25.33 | 23.62 |
|  | Ninghua | 1 | 461 | 4.43 | 21 | 17.58 |
|  | Shanghang | 2 | 215 | 2.11 | 10 | 18.59 |
|  | Qingliu | 2 | 312 | 5.45 | 23.5 | 16.58 |
|  | Guihua | 1 | 366 | 1.39 | 16 | 25.18 |
| Guangzhou | Nanhai | 2 | 14.5 | 3.82 | 9.5 | 20.50 |
|  | Panyu | 2 | 17.5 | 7.54 | 15.5 | 22.46 |
|  | Dongguan | 2 | 7 | 0.75 | 3 | 23.21 |
|  | Sanshui | 1 | 13 | 2.46 | 11 | 21.31 |
|  | Conghua | 1 | 37 | 1.82 | 10 | 24.15 |
|  | Zengcheng | 1 | 3 | 2.88 | 11 | 27.40 |
|  | Qingyuan | 4 | 12 | 1.09 | 7.25 | 16.08 |

Note: Corresponding data for counties not listed in the table is calculated as 0.

**Table S2-2 Spatial data statistics of Diyunsuo in Wenzhou, Tingzhou and Guangzhou**

| **Prefecture** | **County** | **Average elevation**  **（m）** | **Average slope**  **（°）** | **Average relief**  **（m）** | **Average distance**  **（km）** |
| --- | --- | --- | --- | --- | --- |
| Guangzhou | Nanhai | 20 | 2.9 | 15 | 72.33 |
|  | Xinhui | 1 | 0 | 0 | 72.33 |
|  | Qingyuan | 43 | 7.14 | 32 | 88.84 |

Note: Corresponding data for counties not listed in the table is calculated as 0.

**Table S2-3 Spatial data statistics of Jidipu in Wenzhou, Tingzhou and Guangzhou**

| **Prefecture** | **County** | **Number** | **Average elevation**  **（m）** | **Average slope**  **（°）** | **Average relief**  **（m）** | **Average distance**  **（km）** | **Average jurisdiction area**  **（km²）** |
| --- | --- | --- | --- | --- | --- | --- | --- |
| Wenzhou | Yongjia | 21 | 17.19 | 5.06 | 17.38 | 3.90 | 163.58 |
|  | Yueqing | 27 | 35.78 | 7.4 | 28.37 | 3.09 | 86.75 |
|  | Rui-an | 26 | 95.42 | 9.54 | 37.54 | 3.95 | 113.66 |
|  | Pingyang | 30 | 85 | 9.38 | 36.67 | 4.29 | 83.66 |
|  | Taishun | 11 | 363.64 | 13.22 | 51.82 | 4.08 | 191.4 |
| Tingzhou | Changting | 20 | 380.9 | 9.81 | 36.25 | 3.79 | 218.73 |
|  | Ninghua | 20 | 474.65 | 10.47 | 41.25 | 5.11 | 171.11 |
|  | Shanghang | 28 | 310.14 | 11.93 | 47.43 | 4.15 | 160.33 |
|  | Wuping | 12 | 373.83 | 13.32 | 46.5 | 4.49 | 267.85 |
|  | Qingliu | 10 | 449.9 | 13.68 | 56.8 | 5.01 | 210.05 |
|  | Liancheng | 5 | 407.4 | 12.49 | 40.6 | 7.05 | 667.93 |
|  | Guihua | 5 | 403.2 | 9.53 | 29 | 4.61 | 447.81 |
|  | Yongding | 4 | 347.25 | 14.35 | 53.75 | 5.67 | 512.41 |
| Guangzhou | Nanhai | 24 | 15.33 | 4.26 | 15.08 | 3.94 | 110.86 |
|  | Panyu | 24 | 31.54 | 5.44 | 21.08 | 2.79 | 70.09 |
|  | Shunde | 3 | 4.67 | 0.57 | 2.33 | 10.20 | 321.33 |
|  | Dongguan | 39 | 54.95 | 7.45 | 30.33 | 4.77 | 142.58 |
|  | Sanshui | 9 | 8.56 | 3.29 | 11.11 | 3.12 | 154.09 |
|  | Conghua | 10 | 47.1 | 5.04 | 20 | 3.66 | 167.69 |
|  | Xinhui | 17 | 22.88 | 3.18 | 14.53 | 7.66 | 204.23 |
|  | Xinning | 12 | 27.75 | 4.98 | 20.17 | 8.81 | 223.06 |
|  | Xiangshan | 1 | 22 | 3.39 | 11 | 31.15 | 1449.72 |
|  | Zengcheng | 16 | 22.63 | 5.22 | 19 | 4.75 | 114.64 |
|  | Qingyuan | 18 | 24.78 | 3.64 | 16.72 | 4.96 | 231.05 |
|  | Longmen | 7 | 105.43 | 10.02 | 36.14 | 6.94 | 450.06 |
|  | Lianzhou | 8 | 224.75 | 8.62 | 34.13 | 10.76 | 414.87 |
|  | Yangshan | 11 | 341.18 | 16.81 | 59.73 | 6.42 | 464.97 |
|  | Lianshan | 3 | 424.67 | 8.51 | 40.33 | 6.17 | 610.2 |

**2. Dimensionless processing of the data**

The dimensions of the data above are inconsistent and and could not be directly substituted into the evaluation system for calculation. To eliminate this impact, it is necessary to perform a unified dimensionless process to make it comparable.

According to the numerical distribution of each indicator, the values of each indicator were divided into 10 intervals in a hierarchical manner, with corresponding scores ranging from 1 to 10 integer levels, thereby making the attribute value of each evaluation indicator in a unified dimension. This method can unify the positive and negative indicators directly, and take into account the characteristics of the spatial distribution and transmission process of the postal system, as well as eliminating the influence of extreme values. For example, for indicators such as slope, the range of 0-2° is defined as flat land in the land classification standard [], which has similar influence on the accessibility of the postal system, so they can be discussed together. The detailed standards are as follows.

**Number**

The number of Yizhan and Jidipu varies in each county, so it needs to be discussed separately. Statistics show that during the Ming Dynasty, there were a maximum of 5 Yizhan and 44 Jidipu in a county along the southeast coast of China, and the number of Yizhan and Jidipu were divided into intervals proportionally, with the corresponding scores in parentheses, as follows:

Number of Yizhan: 1 (2), 2 (4), 3 (6), 4 (8), 5 (10).

Number of Jidipu: 1~5 (1), 6~10 (2), 11~15 (3), 16~20 (4), 21~25 (5), 26~30 (6), 31~35 (7), 36~40 (8), 41~45 (9), ≥46 (10).

**Elevation**

For elevation, areas with high altitude are generally mountainous and hilly, which is not conducive to the transmission and results in low accessibility. So the numerical increment in elevation is inversely proportional to the score value. According to statistics, most of the postal facilities were located in areas below an elevation of 500m. Therefore, the division of elevation intervals is taken as a unit of 50m, as follows:

Elevation: ＜50m (10), 50~100m (9), 100~150m (8), 150~200m (7), 200~250m (6), 250~300m (5), 300~350m (4), 350~400m (3), 400~450m (2), ≥450m (1).

**Slope**

The increase in slope is not conducive to the transmission process, so its numerical increase shows an opposite trend to the score, with higher slope corresponding to lower score. Regularly, slope of land in China is classified as 2°, so the interval division of slope is taken as a unit of 2°, as follows：

Slope: 0~2° (10), 2~4° (9), 4~6° (8), 6~8° (7), 8~10°（6), 10~12° (5), 12~14° (4), 14~16° (3), 16~18° (2), ≥18° (1).

**Relief**

Similarly, the numerical increase in relief is also inversely proportional to the score. The higher the slope, the greater the obstacle to transmission, and the lower the corresponding score. According to the statistics of relief, most of the postal facilities were located in the area of relief <45m, so the interval division of relief is taken as a unit of 5m, as follows:

0~5m (10), 5~10m (9), 10~15m (8), 15~20m (7), 20~25m (6), 25~30m (5), 30~35m (4), 35~40m (3), 40~45m (2), ≥45m (1).

**Distance**

Normally, the farther the distance between the postal facilities, the longer time the transmission took, and the lower the accessibility. Therefore, the increase of releif is inversely proportional to the score. According to the distance statistics of the postal facilities, the minimum distance between Yizhan was within 10km, and the maximum distance was above 60km. Thus, the interval division of Yizhan’s distance starts from 10km and increases by 5km. The distance between Diyunsuo was large, with a minimum of 10km and a maximum of 100km, so the interval division of Diyunsuo’s distance starts from 10km and increases by 10km. The minimum distance between Jidipu was within 3km, and the maximum distance was above 10km, so its interval division starts from 3km and increases by 1km. The specific score conversion criteria are as follows:

Yizhan: ＜10km (10), 10~15km (9), 15~20km (8), 20~25km (7), 25~30km (6), 30~35km (5), 35~40km (4), 40~45km (3), 45~50km (2), ＞50km (1).

Diyunsuo: ＜10km (10), 10~20km (9), 20~30km (8), 30~35km (7), 35~40km (6), 40~45km (5), 45~50km (4), 50~55km (3), 55~60km (2), ＞60km (1).

Jidipu: ＜2km (10), 2~3km (9), 3~4km (8), 4~5km (7), 5~6km (6), 6~7km (5), 7~8km (4), 8~9km (3), 9~10km (2), ＞10km (1).

**Jurisdiction area**

The larger the area under the jurisdiction of Jidipu, the worse the information flow in remote areas within the area. Thus, the increase of the jurisdiction area is also inversely proportional to the score. Statistics of the Jidipu’s jurisdiction area shows that most of them are in the range of 100~500km². Accordingly, the interval division of jurisdiction area starts from 100km² and increases by 50km², as follows:

＜100km² (10), 100~150km² (9), 150~200km² (8), 200~250km² (7), 250~300km² (6), 300~350km² (5), 350~400km² (4), 400~450km² (3), 450~500km² (2), ＞500km² (1).

According to the above score conversion criteria, the spatial data of the research object were converted and the results are shown in Tables S2-4, S2-5, and S2-6. The obtained values can be substituted into the accessibility evaluation model for calculation.

**Table S2-4 Data conversion result of Yizhan in Wenzhou, Tingzhou and Guangzhou**

| **Prefecture** | **County** | **Number** | **Average elevation** | **Average slope** | **Average relief** | **Average distance** |
| --- | --- | --- | --- | --- | --- | --- |
| Wenzhou | Yongjia | 2 | 10 | 8 | 7 | 9 |
|  | Yueqing | 8 | 10 | 6 | 4 | 8 |
| Tingzhou | Changting | 6 | 3 | 6 | 5 | 7 |
|  | Ninghua | 2 | 1 | 8 | 6 | 8 |
|  | Shanghang | 4 | 6 | 9 | 8 | 8 |
|  | Qingliu | 4 | 4 | 8 | 6 | 8 |
|  | Guihua | 2 | 3 | 10 | 7 | 6 |
| Guangzhou | Nanhai | 4 | 10 | 9 | 9 | 7 |
|  | Panyu | 4 | 10 | 7 | 7 | 7 |
|  | Dongguan | 4 | 10 | 10 | 10 | 7 |
|  | Sanshui | 2 | 10 | 9 | 8 | 7 |
|  | Conghua | 2 | 10 | 10 | 8 | 7 |
|  | Zengcheng | 2 | 10 | 9 | 8 | 6 |
|  | Qingyuan | 8 | 10 | 10 | 9 | 8 |

Note: Corresponding data for counties not listed in the table is calculated as 0.

**Table S2-5 Data conversion result of Diyunsuo in Wenzhou, Tingzhou and Guangzhou**

| **Prefecture** | **County** | **Average elevation** | **Average slope** | **Average relief** | **Average distance** |
| --- | --- | --- | --- | --- | --- |
| Guangzhou | Nanhai | 5 | 9 | 8 | 3 |
|  | Xinhui | 5 | 10 | 10 | 3 |
|  | Qingyuan | 5 | 7 | 4 | 2 |

Note: Corresponding data for counties not listed in the table is calculated as 0.

**Table S2-6 Data conversion result of Jidipu in Wenzhou, Tingzhou and Guangzhou**

| **Prefecture** | **County** | **Number** | **Average elevation** | **Average slope** | **Average relief** | **Average distance** | **Average jurisdiction area** |
| --- | --- | --- | --- | --- | --- | --- | --- |
| Wenzhou | Yongjia | 5 | 10 | 8 | 7 | 8 | 8 |
|  | Yueqing | 6 | 10 | 7 | 5 | 8 | 10 |
|  | Rui-an | 6 | 9 | 6 | 3 | 8 | 9 |
|  | Pingyang | 6 | 9 | 6 | 3 | 7 | 10 |
|  | Taishun | 3 | 3 | 4 | 1 | 7 | 8 |
| Tingzhou | Changting | 4 | 3 | 6 | 3 | 8 | 7 |
|  | Ninghua | 4 | 1 | 5 | 2 | 6 | 8 |
|  | Shanghang | 6 | 4 | 5 | 1 | 7 | 8 |
|  | Wuping | 3 | 3 | 4 | 1 | 7 | 6 |
|  | Qingliu | 2 | 2 | 4 | 1 | 6 | 7 |
|  | Liancheng | 1 | 2 | 4 | 2 | 4 | 1 |
|  | Guihua | 1 | 2 | 6 | 5 | 7 | 3 |
|  | Yongding | 1 | 4 | 3 | 1 | 6 | 1 |
| Guangzhou | Nanhai | 5 | 10 | 3 | 7 | 8 | 9 |
|  | Panyu | 5 | 10 | 1 | 6 | 9 | 10 |
|  | Shunde | 1 | 10 | 8 | 10 | 1 | 5 |
|  | Dongguan | 8 | 9 | 7 | 4 | 7 | 9 |
|  | Sanshui | 2 | 10 | 9 | 8 | 8 | 8 |
|  | Conghua | 2 | 10 | 8 | 6 | 8 | 8 |
|  | Xinhui | 4 | 10 | 9 | 8 | 4 | 7 |
|  | Xinning | 3 | 10 | 8 | 6 | 3 | 7 |
|  | Xiangshan | 1 | 10 | 9 | 8 | 1 | 1 |
|  | Zengcheng | 4 | 10 | 8 | 7 | 7 | 9 |
|  | Qingyuan | 4 | 10 | 9 | 7 | 7 | 7 |
|  | Longmen | 2 | 8 | 5 | 3 | 5 | 2 |
|  | Lianzhou | 2 | 6 | 6 | 4 | 1 | 3 |
|  | Yangshan | 3 | 4 | 2 | 1 | 5 | 2 |
|  | Lianshan | 1 | 2 | 6 | 2 | 5 | 1 |
