# Supplementary material for: Exploring the regional layout characteristics of ancient Chinese postal system in coastal areas based on AHP-CRITIC evaluation approach
Source: PLoS One. 2025 Sep 25;20(9):e0333348. doi: 10.1371/journal.pone.0333348 (PMC12463204; doi:10.1371/journal.pone.0333348)
Supplement: S1 Table — (PDF) [file pone.0333348.s003.pdf]

S1 Table Details of Wenzhou's postal system in the Ming Dynasty

Prefecture	County	Type of facility	Facility	Elevation (m)	slope (°)	Relief (m)	Distance (km)	Jurisdiction area of Jidipu (km²)
Wenzhou	Yongjia	Yizhan	Xiangpu	10	5.05	18	10.21	/
		Jidipu	Master Pu	10	6.02	15	2.72	48.53
			Chengnan	20	3.32	9	3.04	29.64
			Cihu	3	0.00	2	3.21	44.56
			Guanghua	3	0.00	1	2.72	28.86
			Shangshu	3	0.75	2	4.97	158.54
			Xiaodan	5	0.00	4	2.10	763.06
			Guacai	152	27.71	101	5.55	307.29
			Quyu	4	0.95	1	3.89	50.01
			Puzhou	6	1.22	3	3.89	43.53
			Ningcun Suo Qian	3	0.34	2	4.63	26.15
			Xiaxian	5	0.95	4	4.69	164.11
			Lintou	16	12.95	32	4.72	120.76
			Sangxi	10	1.43	10	4.43	124.23
			Jiaoyang	75	23.43	79	2.10	138.02
			Maozhu	6	2.36	6	5.13	56.32
			Nanmen	6	2.88	6	3.84	47.96
			Changsha	3	0.75	3	3.84	53.06
			Xiao Guishan	4	0.95	2	3.69	39.49
			Shangwan	3	1.82	4	4.63	16.45
			Jiangnan	11	8.69	34	4.07	961.33
			Xialong	13	9.76	45	4.07	213.35
	Yueqing	Yizhan	Guantou	6	4.72	31	10.21	/
			Lingdian	58	20.72	64	21.02	/
			Xi-ao	9	3.20	9	18.10	/
			Yao-aoling	7	4.76	25	18.10	/
		Jidipu	Master Pu	15	7.78	31	2.13	78.52
			Baisha	4	0.00	3	2.13	29.99
			Dalin	5	0.00	3	4.32	70.10
			Xinshi	4	0.00	6	3.94	121.27
			Wushi	148	15.95	63	3.23	24.38
			Lanyu	2	0.00	2	5.73	930.88
			Shizhen	54	23.29	92	1.66	19.60
			Dajing	9	2.13	5	2.86	112.13
			Panshan	313	36.47	125	4.98	30.19
			Puqi Suo Qian	3	0.68	2	3.35	22.78
			Changshan	16	17.23	67	3.35	21.98

			Sanjiang	5	1.01	2	3.23	24.43
			Cai-ao	17	8.39	22	1.66	10.46
			Tiaotou	99	30.11	113	2.05	3.74
			Huwu	80	18.22	71	2.88	11.23
			Huhuang	4	0.75	3	3.95	120.79
			Tangxia	6	1.97	4	1.34	141.06
			Shichuan	4	0.75	2	1.34	49.92
			Guantou	9	2.88	8	4.80	44.21
			Panshi Wei Qian	3	1.07	2	4.80	22.71
			Chenggang	5	2.13	4	3.35	63.54
			Huangshan	8	1.91	5	2.05	16.37
			Yitou	23	8.69	38	2.10	74.97
			Pingfeng	3	0.34	3	3.88	12.56
			Xiao Chenggang	4	2.88	23	2.10	4.71
			Xiao Huangshan	115	14.13	58	2.47	8.15
			Tianxian	8	1.07	9	3.80	271.47
	Rui-an	Jidipu	Master Pu	10	3.58	21	3.99	12.01
			Shayuan	6	2.57	6	2.71	46.19
			Feiyun	14	4.11	8	4.15	17.97
			Xiang-ao	5	0.34	6	3.57	61.15
			Tuanyu	5	3.34	5	3.57	162.41
			Shipai	6	3.58	7	4.18	128.71
			Panshan	119	13.17	48	3.90	143.98
			Gexi	275	29.86	114	3.90	228.32
			Wangyu	11	1.22	12	7.40	174.23
			Guanyan	346	8.44	44	3.96	356.35
			Huanglou	242	17.04	82	2.87	124.29
			Dayang	228	25.26	109	4.83	208.99
			Guixi	456	21.46	82	3.69	260.48
			Guishan	6	0.00	2	5.53	68.72
			Dingtian	24	4.50	19	3.56	72.09
			Dongshan	12	5.26	11	3.39	36.37
			Xianju	6	3.47	8	3.40	22.83
			Zi-ao	4	0.95	2	3.21	100.38
			Litang	110	19.05	77	2.65	99.90
			Ma-ao	152	14.13	53	2.65	24.54
			Qianqiao	5	1.07	2	3.88	60.59
			Wuchi	4	0.34	2	4.15	78.73
			Tankun	201	25.21	108	2.87	33.86
			Taihu	131	15.24	59	5.66	93.63
			Huangshan	96	22.61	84	5.66	302.74
			Sizhuang	7	2.13	5	3.39	35.60

	Pingyang	Jidipu	Master Pu	212	14.40	46	5.03	54.95
			Ying-en	5	1.07	2	4.78	39.21
			Wanquan	5	0.75	4	4.20	33.71
			Changshan	7	2.86	9	2.66	63.28
			Pumen	7	0.00	2	2.36	100.26
			Huangxiang	243	10.86	51	4.12	54.10
			Qixi	141	16.47	59	4.12	62.18
			Shuangpai	257	31.54	120	4.67	71.31
			Shitang	108	34.84	174	4.43	64.00
			Yulin	238	20.78	85	7.22	45.63
			Guanyuan	3	0.95	1	7.22	42.62
			Xiakou	3	0.00	2	3.55	64.08
			Jiangkou	8	3.39	8	3.98	67.05
			Mocheng	186	1.01	41	4.20	36.34
			Xiankou	5	0.75	5	4.20	42.36
			Fenshui	284	24.60	81	5.23	415.93
			Lingxi	44	21.43	48	4.16	114.27
			Hengdu	5	0.95	3	3.83	70.50
			Xiaodu	5	2.13	4	3.83	56.53
			Dayi	241	17.04	81	5.23	77.38
			Caidian	368	7.14	54	5.31	150.83
			Tangxia	3	1.69	4	4.26	79.01
			Xichen	12	3.04	12	3.52	119.09
			Xiangkou	7	1.01	4	3.52	109.02
			Sizhou	15	4.53	7	3.47	109.03
			Fenghuo	96	32.46	113	3.47	130.05
			Baoqiao	3	1.69	3	4.43	44.31
			Lusi	3	0.48	2	4.67	41.62
			Houwei	10	9.01	33	2.36	42.11
			Zhuangshi	26	14.59	42	4.78	109.04
	Taishun	Jidipu	Master Pu	556	5.72	23	2.83	269.47
			Hongkou	252	15.75	59	4.93	113.97
			Chikeng	582	14.13	47	4.93	261.87
			Zhoukeng	172	23.05	98	2.83	42.79
			Xiage	156	13.82	59	4.12	246.39
			Lin-ao	431	12.60	51	3.46	97.08
			Fang-ao	141	4.82	18	3.55	88.25
			Shangren	611	14.52	60	6.23	214.36
			Chendai	637	19.73	63	4.95	479.07
			Fangcun	212	18.59	69	3.46	132.96
			Baishuiji	250	2.72	23	3.55	159.18
