# Supplementary material for: Exploring the regional layout characteristics of ancient Chinese postal system in coastal areas based on AHP-CRITIC evaluation approach
Source: PLoS One. 2025 Sep 25;20(9):e0333348. doi: 10.1371/journal.pone.0333348 (PMC12463204; doi:10.1371/journal.pone.0333348)
Supplement: S2 Table — (PDF) [file pone.0333348.s004.pdf]

S2 Table Details of Tingzhou's postal system in the Ming Dynasty

Prefecture	County	Type of facility	Facility	Elevation (m)	slope (°)	Relief (m)	Distance (km)	Jurisdiction area of Jidipu (km²)
Tingzhou	Changting	Yizhan	Linting	314	3.11	9	22.01	/
			Guanqian	505	23.06	62	21.79	/
			Sanzhou	263	0.75	5	27.07	/
		Jidipu	Master Pu	314	1.69	8	3.41	573.31
			Xinqiao	373	12.65	67	2.64	154.31
			Yuanyang	382	12.69	63	2.64	105.76
			Hukeng	540	16.48	43	2.85	150.13
			Qiliang	569	11.18	56	2.78	40.65
			Huamei	365	9.06	35	4.34	49.93
			Huangguan	329	6.42	25	5.16	373.78
			Nanjia	295	4.50	16	5.95	139.33
			Hetian	316	9.57	36	5.71	397.38
			Datan	273	4.68	12	4.38	100.23
			Sanzhou	281	11.20	35	4.38	128.81
			Chetian	276	11.49	32	3.61	445.25
			Shuikou	257	2.36	4	3.61	659.19
			Datian	314	8.81	30	4.82	114.49
			Changqiao	360	10.40	39	2.88	273.98
			Shexin	484	11.24	47	2.88	369.00
			Baibu	461	25.14	88	3.41	99.69
			Nantian	336	6.72	19	4.71	118.89
			Daxi	543	4.97	16	2.85	52.76
			Guiren	550	14.92	54	2.78	27.72
	Ninghua	Yizhan	Shiniu	461	4.43	21	17.58	/
		Jidipu	Master Pu	332	12.82	33	6.63	576.06
			Yulong	374	4.82	31	5.47	69.26
			Laishe	450	17.29	67	3.80	134.66
			Shiniu	474	11.63	34	2.88	23.94
			Luoxi	474	1.72	8	3.12	83.51
			Huashi	505	8.41	37	3.12	193.27
			Xiafang	488	6.46	35	3.25	74.53
			Huangbai	451	14.35	51	2.83	30.28
			Anle	493	17.10	67	2.83	152.35
			Yangmei	606	16.74	66	2.63	37.88
			Huangdi	506	16.49	60	2.63	247.32
			Xiasha	357	8.98	32	8.47	245.25
			Shaping	458	2.70	19	8.47	263.79
			Yangjia	581	15.72	51	9.99	246.08
			Anyuan	399	1.91	8	9.99	274.38
			Huangyuan	332	8.22	25	5.47	75.23

			Shijing	690	7.14	33	3.25	58.70
			Beiling	561	9.79	56	2.88	78.59
			Zhangdi	588	7.45	52	3.99	144.03
			Shixi	374	19.74	60	10.46	412.99
	Shanghang	Yizhan	Pingxi	214	2.39	8	18.59	/
			Lanwu	216	1.82	12	18.59	/
		Jidipu	Master Pu	190	3.02	8	4.75	64.86
			Daqi	235	12.23	34	2.74	58.97
			Yantou	291	15.87	47	4.33	106.90
			Juanlong	185	1.22	7	2.74	31.06
			Jiantou	200	9.47	52	5.12	37.06
			Yueyang	203	11.35	50	3.83	50.06
			Dawan	251	11.49	32	3.72	26.11
			Shanhu	245	22.86	82	3.21	81.42
			Jiuhua	241	17.85	56	3.21	73.28
			Lanwu	248	4.52	23	1.77	84.75
			Guanzhuang	285	9.52	43	1.77	188.33
			Huilong	302	14.07	50	5.63	422.36
			Jiutai	408	8.69	35	1.95	46.82
			Shuangxi	425	19.48	57	2.85	75.57
			Denglong	416	22.90	81	2.85	121.34
			Shiming	414	11.01	55	4.83	1071.45
			Anxiang	273	18.52	57	5.30	117.74
			Lufeng	267	4.58	23	5.30	218.33
			Eling	604	31.43	115	5.31	105.74
			Meixi	258	17.49	72	7.54	99.87
			Fangcun	377	8.28	49	3.86	42.85
			Dingta	394	4.68	38	4.18	48.46
			Zhangqing	440	4.76	28	4.18	75.17
			Chatian	389	2.36	22	1.95	57.91
			Tangbian	496	19.38	82	4.83	166.61
			Bianmin	248	13.93	59	3.86	149.59
			Guiren	183	4.38	22	5.31	190.68
			Niuping	216	8.77	49	9.33	675.89
	Wuping	Jidipu	Master Pu	256	8.84	27	5.44	619.76
			Sanjiao	390	15.07	42	4.59	167.74
			Zhongtian	427	18.25	72	4.59	320.76
			Huangbai	228	6.06	17	4.59	131.43
			Yuanshe	525	18.50	68	4.24	154.08
			Laiwu	413	19.18	78	4.59	190.89
			Huangjing	502	14.87	50	4.63	142.72
			Daling	557	6.02	29	4.24	195.99
			Jiaodong	354	17.97	66	4.17	85.33
			Zaojiao	311	9.76	24	3.65	46.51
			Tanling	219	16.70	62	3.65	24.02

			Wu-an	304	8.61	23	5.44	1134.96
	Qingliu	Yizhan	Yuhua	321	5.14	18	16.58	/
			Jiulong	303	5.76	29	16.58	/
		Jidipu	Master Pu	475	25.50	107	2.80	35.95
			Leigong	430	16.31	69	3.76	53.12
			Xiaosong	371	17.78	66	4.93	473.03
			Songxi	344	8.06	29	5.31	286.74
			Zisun	628	1.69	32	4.46	275.13
			Wujia	513	35.95	125	2.80	65.07
			Xuanshui	332	6.85	19	8.73	196.66
			Wudi	363	14.35	57	5.31	161.17
			Yuhua	546	4.76	36	5.47	293.53
			Taiping	497	5.55	28	5.47	260.05
	Liancheng	Jidipu	Master Pu	349	8.08	20	6.14	2160.48
			Bei-an	381	4.82	14	6.14	459.42
			Fenshui	431	20.52	86	6.50	310.34
			Xintian	353	9.09	32	7.09	267.76
			Nanchai	523	19.96	51	6.73	141.63
	Guihua	Yizhan	Mingxi	366	1.39	16	25.18	/
		Jidipu	Master Pu	332	3.72	7	4.92	270.19
			Longhu	471	18.37	59	5.54	1095.64
			Yushan	357	2.16	8	5.19	338.72
			Xinyu	405	2.16	18	3.70	217.49
			Yong-an	451	21.21	53	3.70	317.00
	Yongding	Jidipu	Master Pu	225	26.52	94	4.74	1292.84
			Jiejing	532	10.78	42	5.03	289.45
			Dehua	311	5.79	28	4.74	145.78
			Xingan	321	14.31	51	7.81	321.56
