# Supplementary material for: Exploring the regional layout characteristics of ancient Chinese postal system in coastal areas based on AHP-CRITIC evaluation approach
Source: PLoS One. 2025 Sep 25;20(9):e0333348. doi: 10.1371/journal.pone.0333348 (PMC12463204; doi:10.1371/journal.pone.0333348)
Supplement: S3 Table — (PDF) [file pone.0333348.s005.pdf]

S3 Table Details of Guangzhou's postal system in the Ming Dynasty

Prefecture	County	Type of facility	Facility	Elevation (m)	slope (°)	Relief (m)	Distance (km)	Jurisdiction area of Jidipu (km²)
Guangzhou	Nanhai	Yizhan	Xujiang	25	5.27	12	20.24	/
			Guanyao	4	2.36	7	20.77	/
		Diyunsuo	Guangzhou	20	2.90	15	72.33	/
		Jidipu	Master Pu	13	3.77	19	3.40	85.83
			Longtang	18	11.40	45	1.36	47.51
			Anxi	9	4.17	11	2.74	34.36
			Yuntai	18	4.38	9	2.88	28.87
			Shima	14	5.26	14	2.88	27.82
			Guanfeng	16	4.26	13	3.21	56.22
			Maoshan	20	4.39	14	3.51	169.40
			Yichi	9	1.07	4	4.46	53.33
			Hengtan	5	2.78	11	3.24	258.63
			Lingjiang	3	2.72	6	3.24	74.01
			Tongguang	18	3.52	13	4.10	98.42
			Xinyao	118	13.41	58	3.69	130.06
			Niulan	22	3.47	18	3.69	118.82
			Yandong	10	0.75	5	3.78	141.43
			Shuidong	3	1.07	8	2.73	113.83
			Fu-nan	16	5.40	26	3.68	48.72
			Huabiao	14	3.11	11	4.82	87.68
			Zitong	5	2.16	9	4.96	122.50
			Shipai	3	2.26	8	4.15	75.38
			Dakeng	6	0.00	5	5.98	86.61
			Xiaodong	6	1.35	5	6.03	141.45
			Shabian	3	2.70	4	5.95	166.65
			Shakeng	6	9.21	24	5.95	288.30
			Wudoukou	13	9.63	22	4.15	204.81
	Panyu	Yizhan	Wuyang	19	10.18	24	20.77	/
			Panhu	16	4.90	7	24.15	/
		Jidipu	Songbai	32	4.73	13	2.94	73.43
			Kaiqizheng	49	5.32	20	1.36	14.44
			Kaiqizhong	102	19.78	72	2.74	17.29
			Modaokeng	112	3.34	33	2.82	32.23
			Henggangzheng	96	27.71	101	2.46	39.10
			Henggangzhong	20	6.09	18	2.46	26.42
			Niujingling	19	4.04	19	3.02	52.26
			Lengshuikeng	29	2.88	15	2.65	67.56
			Jiyazheng	78	13.07	54	2.31	65.90
			Jiyazhong	34	8.94	26	2.31	56.01
			Jiya Xia	30	1.43	12	1.98	83.92

			Meitian	24	2.26	13	1.98	64.92
			Kangtongzhong	14	4.50	18	2.99	94.67
			Songbaitang	21	6.85	13	2.99	97.46
			Yangshuzheng	27	2.86	9	3.28	89.82
			Dashuizheng	20	10.48	27	3.40	22.73
			Dashuizhong	29	4.07	21	3.51	46.51
			Chebeizheng	5	0.00	5	3.51	60.53
			Chebeizhong	3	0.00	5	3.06	81.55
			Baishizheng	2	1.07	2	3.06	95.34
			Baishizhong	3	0.00	1	3.05	107.71
			Baimang	4	1.07	6	2.97	114.25
			Wushizheng	1	0.00	0	2.97	102.19
			Wushizhong	3	0.00	3	3.17	175.97
	Shunde	Jidipu	Master Pu	7	0.95	3	9.83	225.52
			Dazhou	5	0.00	2	9.83	275.91
			Dunian	2	0.75	2	10.95	462.56
	Dongguan	Yizhan	Tiegang	8	0.75	3	23.21	/
			Huangjiashan	6	0.75	3	23.21	/
		Jidipu	Master Pu	21	5.73	14	4.29	118.58
			Jiaoli	3	1.01	4	5.68	146.36
			Dugang	7	1.43	6	4.34	68.33
			Baima	11	2.05	10	4.14	65.22
			Hetian	12	5.48	15	6.92	150.89
			Shenxi	31	4.07	20	5.19	65.92
			Chigang	6	0.75	3	3.69	64.08
			Yangwanwei	28	3.65	9	3.69	87.89
			Dujian	12	0.75	8	4.37	68.77
			Tutian	5	0.00	2	4.37	84.99
			Chengdong	8	4.68	9	5.01	337.40
			Yancun	97	15.21	55	5.42	246.00
			Shuanmen	57	5.81	31	4.78	69.86
			Baisha	36	2.90	22	4.78	153.39
			Heshanggang	6	1.39	8	4.95	46.33
			Xinyong	5	2.43	4	4.95	34.45
			Nitang	94	27.64	99	6.79	118.62
			Dayong	2	0.34	4	5.27	72.14
			Yangshujiao	30	2.05	11	5.16	88.92
			Shawei	12	7.45	39	5.16	330.47
			Shangbu	11	0.34	6	2.43	160.54
			Yuegang	15	1.35	9	2.43	192.69
			Luolidong	112	11.37	53	4.15	206.20
			Pengkeng	426	32.10	143	4.14	166.05
			Gulou	227	8.11	37	4.12	153.75
			Yantian	54	12.05	73	4.12	460.93
			Shangmeisha	23	2.72	21	2.79	61.34

			Xiameisha	149	15.94	51	2.79	15.01
			Xiyong	59	30.55	100	3.47	36.31
			Xiadong	98	15.24	63	3.47	27.18
			Aotou	109	19.86	66	4.44	32.21
			Diefu	23	2.70	7	2.54	33.03
			Wuyong	6	0.34	2	1.07	35.33
			Dapeng	5	0.75	3	1.07	52.26
			Dalu	252	29.50	135	15.40	250.58
			Huangshashui	11	3.44	11	9.49	739.52
			Maotian	39	4.29	13	6.93	85.98
			Yingcun	4	1.69	9	4.14	37.26
			Zhoujiacun	37	3.32	8	7.98	395.70
	Sanshui	Yizhan	Xi-nan	13	2.46	11	21.31	/
		Jidipu	Master Pu	17	5.05	15	6.16	295.60
			Liantang	2	0.00	2	6.16	617.29
			Xian-gang	11	1.82	7	2.70	206.13
			Huashan	6	4.11	11	2.70	88.16
			Xian-an	5	3.72	11	2.66	35.14
			Qingshui	13	3.44	11	2.48	42.21
			Wangxian	3	4.07	17	1.61	21.67
			Yangmei	16	3.02	14	1.61	27.57
			Zhuogui	4	4.38	12	2.02	53.07
	Conghua	Yizhan	Lishiqi	37	1.82	10	24.15	/
		Jidipu	Master Pu	27	2.78	12	5.90	904.14
			Niuwangzhong	18	3.04	11	3.82	91.73
			Huangzhukeng	29	2.26	9	3.82	82.97
			Jintou	52	15.75	40	3.40	53.09
			Foziling	20	0.48	9	3.35	78.23
			Niuwangzheng	40	3.32	17	4.35	48.89
			Xiangling	125	4.79	33	2.35	56.02
			Dawolang	78	11.31	52	2.35	60.88
			Xintian	50	4.26	10	4.06	102.29
			Yeyatang	32	2.39	7	3.22	198.67
	Xinhui	Diyunsuo	Xinhui	1	0.00	0	72.33	/
		Jidipu	Master Pu	2	0.34	1	4.49	56.48
			Fengfeng	19	2.57	9	5.30	177.43
			Dayuan	17	1.72	6	5.01	157.43
			Hengcha	3	2.64	18	5.01	172.70
			Chaoxian	6	1.22	5	5.54	151.31
			Gucan	83	7.80	33	5.54	62.52
			Poting	50	4.39	31	7.26	192.85
			Liantang	8	4.68	22	7.86	115.54
			Qiaoting	7	2.90	8	8.91	205.06
			Caoping	14	1.35	5	8.77	117.42
			Guanlai	2	1.39	2	4.49	221.29

			Qiaoting	126	12.96	55	8.22	209.53
			Xiangang	6	0.34	1	9.55	304.69
			Liubian	17	1.69	16	10.47	531.18
			Ma-an	5	5.13	21	10.83	227.56
			Dulu	6	0.00	1	13.14	396.18
			Bangchong	18	3.02	13	9.92	172.66
	Xinning	Jidipu	Master Pu	16	7.39	17	6.21	203.14
			Jitang	4	8.08	9	6.59	133.84
			Li-ao	35	1.82	25	6.59	89.86
			Shanbei	3	1.22	9	6.21	210.31
			Licun	76	12.34	58	9.46	220.30
			Nagen	9	2.90	9	9.45	222.18
			Gulong	4	0.48	2	9.45	278.60
			Shuangmen	44	16.67	72	10.30	188.77
			Songjing	6	3.20	7	10.30	172.28
			Luma	1	1.07	5	12.61	261.68
			Hengshan	16	1.01	7	9.28	362.73
			Nanhua	119	3.58	22	9.28	333.08
	Xiangshan	Jidipu	Master Pu	22	3.39	11	31.15	1449.72
	Zengcheng	Yizhan	Dongzhou	3	2.88	11	27.40	/
		Jidipu	Zengjiang	40	12.64	47	3.17	44.04
			Fenghuangzheng	33	16.18	61	3.17	19.21
			Fenghuangzhong	19	6.89	18	3.65	70.09
			Hezi	17	4.52	15	3.76	19.37
			Kelu	17	1.69	13	3.94	45.71
			Quanjing	5	0.75	2	4.50	81.67
			Ehu	3	1.07	3	3.84	68.18
			Nietian	11	2.13	5	3.84	79.19
			Hetang	11	4.58	11	4.11	112.24
			Foling	13	3.93	12	4.34	74.52
			Liantang	21	5.32	15	6.95	152.90
			Daowei	20	5.10	24	6.92	232.97
			Dingtang	40	2.72	17	6.92	331.67
			Duntou	10	1.01	6	3.88	44.50
			Shapu	67	8.74	43	6.44	92.22
			Puxu	35	6.26	12	6.66	365.76
	Qingyuan	Yizhan	Hengshi	6	0.00	4	13.62	/
			Anyuan	20	2.88	13	18.55	/
			Guanzhuang	9	0.48	8	13.62	/
			Huiqi	13	1.01	4	18.55	/
		Diyunsuo	Hengshi	43	7.14	32	88.84	/
		Jidipu	Master Pu	22	2.72	10	8.44	1234.57
			Shantang	3	0.95	2	8.27	644.06
			Matong	5	0.34	8	8.27	247.06
			Qingshi	29	10.68	43	10.29	203.77

			Taiping	7	1.91	10	6.91	413.89
			Xiatou	60	10.48	39	6.11	229.09
			Renkeng	59	3.65	26	5.84	152.90
			Hengshi	3	2.13	14	4.34	54.26
			Datang	15	3.93	13	3.03	19.37
			Beikeng	51	6.53	25	2.97	30.45
			Xia-e	21	1.72	9	2.97	56.86
			Nankeng	28	1.22	11	2.90	38.08
			Dutou	20	0.48	8	2.90	74.83
			Wushi	26	1.69	22	3.22	84.89
			Damiao	30	5.81	22	3.13	346.68
			Baishi	15	1.82	8	3.13	83.68
			Danzhu	12	2.88	10	3.33	140.04
			Baitu	40	6.53	21	3.22	104.48
	Longmen	Jidipu	Ganxiang	64	1.35	4	4.92	513.92
			Huangxi	97	7.59	23	4.92	329.51
			Yancun	191	19.03	73	7.94	382.58
			Changtan	54	11.08	38	8.72	456.28
			Youtian	51	10.85	39	7.65	839.56
			Chifo	260	18.33	69	7.23	250.92
			Xiakou	21	1.91	7	7.23	377.61
	Lianzhou	Jidipu	Master Pu	141	5.01	21	10.54	253.74
			Shiquan	108	2.46	12	10.84	551.02
			Guanzi	375	21.91	83	7.67	357.52
			Dongshan	510	19.05	65	8.24	220.34
			Fengmen	201	3.11	33	7.64	102.19
			Dalong	197	6.93	25	11.91	796.52
			Shunping	136	4.26	15	11.91	569.24
			Lianqian	130	6.26	19	17.33	468.35
	Yangshan	Jidipu	Master Pu	105	13.83	54	5.29	1317.76
			Dongzhai	160	12.30	41	5.29	431.69
			Chaoshui	487	27.58	75	5.60	116.10
			Damu	532	28.14	133	6.35	277.59
			Xiaping	347	13.82	53	6.35	326.70
			Shengshui	553	20.85	62	6.45	275.42
			Zhatian	362	17.65	41	7.02	178.68
			Niumian	390	13.81	43	7.64	733.32
			Xiangjiang	287	7.97	23	6.75	245.18
			Qinglian	251	16.97	65	6.75	296.44
			Kuangjia	279	12.02	67	7.09	915.81
	Lianshan	Jidipu	Master Pu	282	5.76	17	6.12	1431.32
			Shangji	465	14.25	70	6.12	197.21
			Zhamu	527	5.52	34	6.28	202.07
